# Improved efficiency of two-step amplicon PCR using an acoustic liquid handler

**DOI:** 10.1099/mic.0.001579

**Published:** 2025-07-22

**Authors:** Brooke R. Benz, Eglantina Lopez-Echartea, Briana K. Whitaker, Thomas Baldwin, Barney A. Geddes

**Affiliations:** 1 Department of Plant Pathology, North Dakota State University, Fargo, ND, USA; 2 Department of Microbiological Sciences, North Dakota State University, Fargo, ND, USA; 3 United States Department of Agriculture, Agricultural Research Service, National Center for Agricultural Utilization Research, Mycotoxin Prevention & Applied Microbiology Research Unit, 1815 N University St., Peoria, IL 61604, USA

**Keywords:** amplicon sequencing, automation, microbial ecology, microbiome, miniaturization

## Abstract

The improvement in next-generation sequencing technologies has reduced the costs of sequencing significantly. However, library preparation costs for amplicon sequencing have remained largely unchanged – which is ultimately the cost-limiting step in processing large numbers of microbiome samples. Acoustic liquid handlers can transfer volumes as low as 2.5 nl and have been used to miniaturize several different molecular and cellular assays, including single-step PCR amplicon library preparations. However, there are no current methods available for a two-step library preparation process using an acoustic liquid handler. In this study, we tested the efficiency of an acoustic liquid handler to automate the PCRs and library quantification while also incorporating automated library bead cleanup. We compared the material usage and costs for library preparation and sequencing results of this automated method to the standard, manual method. The automated protocol was able to reduce both PCR reaction volumes fivefold and increased efficiency for library preparation by ~32% without affecting bacterial community compositions. The associated increase in the efficiency of our automated method will allow for greater throughput in sequencing hundreds of microbiome samples without affecting the quality of those sequences.

**Impact Statement**

Library preparation for amplicon sequencing in microbiome studies remains a significant cost constraint, even while sequencing costs have decreased in the last few decades. Acoustic liquid handling robots have been utilized to miniaturize several different assays, including a one-step PCR library preparation process, to reduce costs. However, there are currently no methods available for utilizing acoustic liquid handlers for two-step PCR library preparation. Here, we show that the incorporation of an acoustic liquid handler and automated purification instruments into a two-step library preparation protocol led to a reduction in costs and time to prepare libraries. This method is comparable to the standard manual method and will lead to significant cost savings for the preparation of hundreds of samples required for microbiome studies.

## Data Availability

All protocols, sequence data and analysis codes have been made publicly available in open-access repositories. The authors confirm that all supporting data, code and protocols have been provided within the article or through supplementary data files.

Sequencing data was deposited in the NCBI SRA under the BioProject PRJNA1190462. Accession numbers are available in Table S2 (available in the online Supplementary Material).

R scripts used for data analysis are available at https://github.com/NDSU-Geddes-Lab/brb-libprep doi: 10.5281/zenodo.14194699.

Protocols for automated library preparation have been published on protocols.io and are available at dx.doi.org/10.17504/protocols.io.e6nvwb5r2vmk/v2 and dx.doi.org/10.17504/protocols.io.e6nvwb5w2vmk/v2.

## Introduction

There has been a significant increase in microbiome studies across many systems due to the development of next-generation sequencing (NGS) technologies. These studies aim to characterize the microbiomes of humans [1], plants [2,3], animals [4,5] and the environment [6] ranging from soil [7,8] to marine [9] ecosystems. NGS has made it possible to sequence hundreds to thousands of samples. Since the development of NGS technologies, there has been a significant reduction in sequencing costs, improvement in sequencing quality and increased sample read depth per run [10]. While sequencing capacity and accessibility have greatly improved, there is still a significant bottleneck associated with user time as well as materials and expenses for the library preparation of these samples [11]. Even with an overall reduction in sequencing expenses, performing microbiome studies still requires a substantial investment, with prices for library preparation ranging from ~$30 to $80 per sample [12,13]. To efficiently process these samples, minimizing reaction volumes, reducing reagent usage and preparation time and utilizing high-throughput methods are needed.

Hundreds to thousands of samples are required to characterize microbiomes. However, the significant expense to prepare libraries can limit the sample size in these studies. Technological advancements, like the development of automated pipette-based liquid handlers and acoustic liquid handlers that enable the transfer of small volumes, have allowed for the miniaturization of many processes. Acoustic liquid handlers differ from automated pipette-based liquid handlers in that they use sound waves to transfer precise volumes, thus further saving on pipette tip waste. In this study, we evaluated the efficiency and reproducibility of library preparation in nano-volume reactions using an acoustic liquid handler [14]. Miniaturization of different assays through acoustic liquid handling has successfully been incorporated into a variety of different laboratory processes, including drug discovery and dosage testing [15], MS [16], plasmid sequencing [17], quantitative real-time PCR [18], antigen isolation [19] and cell-free protein synthesis [20]. Thus, acoustic liquid handlers can be incorporated into a variety of different laboratory processes.

Acoustic liquid handlers were previously used to miniaturize amplicon library preparation as part of the Earth Microbiome Project (EMP) protocol [21]. In this protocol, a one-step PCR preparation was performed and compared with the standard protocol involving pipette-based liquid handler (i.e. the Eppendorf epMotion). Reducing the reaction volumes, reagents, pipettes and other consumables significantly decreased the costs of amplicon library preparation. However, the Illumina standard protocol for amplicon library preparation uses a two-step PCR process [22]. The two-step library preparation method involves two separate PCR steps: an amplicon PCR and an index PCR. The amplicon PCR generates amplicons by targeting specific regions such as the V4 region of the 16S rRNA gene for bacteria or the ITS1 region of the rRNA intergenic region for fungi. Next, the index PCR adds unique barcode sequences and Illumina sequencing adapters to the amplicons. It has been shown that this two-step preparation method is more accurate and reduces primer bias [23]. Additionally, there has also been growing interest in using a second set of barcodes during the first round of PCR to increase the number of samples in a single sequencing run [24]. Thus, the two-step PCR approach is likely more amenable to greater throughput than the one-step approach.

In this study, we explored the incorporation of an acoustic liquid handler into the standard two-step library preparation workflows and developed a new protocol for its use. The protocol was applied to a set of microbiome samples from the plant phyllosphere that represent a complex, heterogeneous sample set due to the low microbial loads and the need to block plant organelle DNA with peptide nucleic acids (PNAs) [25,26]. We compared our newly developed protocol to the manual preparation and tracked the efficiency of the usage of consumables, reagents and user time for each step. We also compared the performance of the automated method when sequenced on an Illumina MiSeq versus Illumina NextSeq to compare sequencing results given the expected read depth disparities. Ultimately, the automated protocol led to a significant reduction in costs and user time for library preparation compared with the manual protocol.

## Methods

### Sampling and sample preparation

As a test set for this protocol, we utilized samples collected from a project investigating the impact of *Fusarium* head blight (FHB) disease on the barley microbiome. Ten breeding lines were randomly selected from the USDA-ARS breeding lines in the Aberdeen malting barley training population (Fig. S1A). All ten lines were planted in 2022 at each of three misted FHB nurseries located in Fargo, ND; St. Paul, MN; and Ithaca, NY (Fig. S1B).

FHB symptoms typically develop 2–4 weeks post-flowering; therefore, samples were collected 3–5 weeks after flowering for each location. Five spikes showing visible disease symptoms (labelled ‘Diseased’) and five spikes showing <30% visible symptoms (labelled ‘Non-diseased’) were collected for each genotype. Individual spikes were cut from the stem and then transported in plastic bags amended with Drierite desiccant granules and kept on ice. Samples were stored at −20 °C until further processing.

The spikes were placed in a freeze dryer (Labconco, Kansas City, MO, USA) for 48 to 72 h. Each spike was then individually ground using a laboratory mill grinder (Perten Instruments, Shelton, CT, USA). To minimize cross-contamination, samples of the same genotype and disease state were ground sequentially. Between each spike, the grinder was cleaned by brushing and vacuuming the leftover ground spike material. Between treatments, the grinder was also cleaned with ethanol. The ground material of each spike was collected in individual 15 ml conical tubes and stored at −20 °C.

DNA extractions were performed on 100 mg of ground spike material using 96-well DNA extraction kits (Norgen Biotek, Thorold, Ontario, Canada). Each plate contained three controls (DNA extraction negative, PCR negative and positive control [ZymoBIOMICS Microbial Community Standard, Irvine, CA, USA)]. DNA concentrations were measured with Quant-iT PicoGreen dsDNA Assay Kits (Invitrogen, Waltham, MA, USA) and a BioTek Cytation 5 (Agilent Technologies, Santa Clara, CA, USA) and then diluted to 5 ng µl^−1^.

### Bacterial 16S rRNA amplicon library preparation comparison

**Fig. 1.**
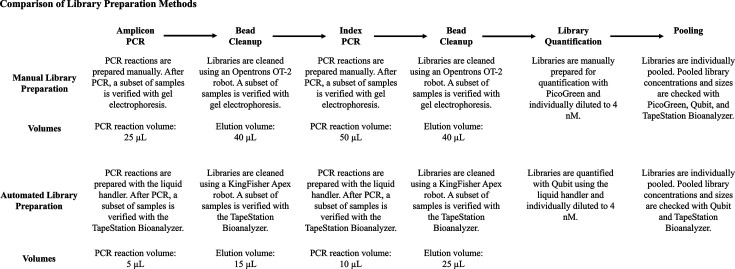
The automated library preparation protocol miniaturized and increased the throughput of library preparation. Comparison of steps between manual and automated library preparation.

A random subset of the field samples described above, amounting to one 96-well plate of samples, was prepared using both methods. Fig. 1 depicts a comparison of the steps involved in the manual and automated library preparation protocols. Broadly, a two-step PCR approach was performed on the V4 region of the 16S rRNA gene to profile bacterial communities. Following conventional practices [21], appropriate checks for successful PCR reactions and amplicon size were made on a random subset of samples for both the manual and automated methods using gel electrophoresis and TapeStation (Agilent Technologies), respectively.

The manual amplicon library preparation was performed following a modified version of the Illumina ‘16S Metagenomic Sequencing Library’ protocol [22]. Individual amplicon PCR reactions consisted of a total reaction volume of 25 µl with 2.5 µl of each forward and reverse V4 amplicon primers (final concentration 0.2 µM; Integrated DNA Technologies, Coralville, IA, USA), 12.5 µl of KAPA HiFi DNA polymerase (final concentration 1×; Roche Diagnostics, Indianapolis, IN, USA), 2.5 µl of each mPNA and pPNA blockers (final concentration 2 µM; PNABio, Inc., Thousand Oaks, CA, USA) and 2.5 µl of template DNA. Nextera primer sequences for V4 16S amplification were: V4_515F: TCGTCGGCAGCGTCAGATGTGTATAAGAGACAGGTGCCAGCMGCCGCGGTAA; V4_R: GTCTCGTGGGCTCGGAGATGTGTATAAGAGACAGGACTACHVGGGTATCTAATCC. Amplicon PCR was performed on a thermocycler (Mastercycler epGradient Eppendorf, Enfield, CT, USA) using the following programme: 95 °C for 3 min; 28 cycles of 95 °C for 30 s, 55 °C for 30 s and 72 °C for 30 s; 72 °C for 5 min; and hold at 4 °C. Libraries were cleaned with 20 µl of Mag-Bind TotalPure NGS magnetic beads (Omega Bio-tek, Inc., Norcross, GA, USA) using an Opentrons OT-2 robot (Opentrons Labworks, Long Island City, NY, USA). The Opentrons robot was programmed to perform bead cleanup following the procedure in the ‘16S Metagenomic Sequencing Library’ protocol [22].

Next, index PCR consisted of a total reaction volume of 50 µl and was performed with 5 µl of each forward and reverse index primers (final concentration 0.1 µM; Nextera primer sequences, Integrated DNA Technologies), 25 µl of DNA polymerase (final concentration 1×), 10 µl of water and 5 µl of template DNA. Index PCR was performed on a thermocycler using the same programme as for amplicon PCR, except 11 cycles instead of 28 cycles. Magnetic bead cleanup was completed after index PCR on the Opentrons robot with the same steps as before, except 56 µl of beads were added to each sample. Libraries were quantified using Quant-iT PicoGreen dsDNA Assay Kits (Invitrogen), diluted to 4 nM and pooled together for sequencing.

The automated library preparation method followed the same basic steps in the library preparation process but with a focus on automation and reduced sample volumes. First, the total reaction volume for amplicon PCR was 5 µl. However, all PCR reagents were included at the same final concentrations, including the template DNA. In the automated method, the mastermix and template DNA were transferred to a 384-well PCR plate using the Echo 525 acoustic liquid handler (Beckman Coulter, Indianapolis, IN, USA) instead of pipettes. Amplicon PCR was performed with an Applied Biosystems QuantStudio 6 Flex Real-Time PCR machine (Invitrogen) using the following programme: 95 °C for 3 min; 30 cycles of 95 °C for 30 s, 55 °C for 30 s and 72 °C for 30 s; 72 °C for 5 min; and hold at 4 °C. An additional two cycles were performed to ensure sufficient amplification with the smaller DNA template volume added. Instead of bead cleanup with the Opentrons robot, individual libraries were cleaned using the KingFisher Apex (Thermo Fisher Scientific, Waltham, MA, USA) as follows. A 96-well sample plate was prepared by combining 10 µl of Mag-Bind TotalPure NGS magnetic beads, 5 µl of molecular-grade water and the libraries. To clean the libraries, two 96-well wash plates were prepared by adding 30 µl of freshly prepared 70% EtOH. To elute the cleaned libraries, 15 µl of molecular-grade water was added to a 96-well elution plate.

Next, the Echo 525 liquid handler was used to transfer the index PCR mastermix and indices to a 384-well PCR plate. Template DNA was manually added to each well. Individual index PCR reactions consisted of 1 µl each of forward and reverse Nextera indices, 5 µl of DNA polymerase, 1 µl of molecular-grade water and 2 µl of template DNA, for a total reaction volume of 10 µl. Index PCR was performed with an Applied Biosystems QuantStudio 6 Flex Real-Time PCR machine using the same programme as the manual method. The index PCR was kept at 10 µl to ensure a sufficient concentration after bead cleanup. The samples were cleaned using the KingFisher Apex as before. The elution plate had 25 µl of molecular-grade water, and the sample plate had 10 µl of magnetic beads. Libraries were quantified with Invitrogen Qubit 1X HS (Invitrogen) and the BioTek Cytation 5 (Agilent Technologies) instead of Quant-iT PicoGreen for the miniaturization of library quantification using the Echo 525 liquid handler, which transferred 0.6 µl of each library, while 61.4 µl of the Qubit dsDNA HS working solution buffer was added manually using a multichannel pipette. Libraries were diluted to 4 nM and pooled per preparation method.

### Library quantification comparison

Libraries prepared with the manual method were quantified manually using Quant-iT PicoGreen assays, while libraries prepared with the automated method were quantified with Invitrogen Qubit 1X HS using the Echo 525 liquid handler. Because of the differences in quantification methods, the library DNA concentrations were correlated through linear regression.

### Sequencing

All samples were first sequenced on a single 250 PE v2 MiSeq run (Illumina, San Diego, CA, USA) with a 10% PhiX spike-in. In addition, samples prepared with the Echo 525 acoustic liquid handler were also sequenced on a NextSeq 1000 (Illumina) using a P1 standard cartridge with a 40% PhiX spike-in.

### Data analysis

All bioinformatic processing and statistical analyses were performed in R (v. 4.1.3) [27]. Sequences were assessed for quality, and amplicon sequencing variants (ASVs) were generated using DADA2 (v. 1.26.0) [28]. Low-quality reads, chimaeras, chloroplast, mitochondrial and Eukaryota reads were removed. Read counts for each method were tracked throughout sequence processing, and total read depth, average read depth and high and low read depths were calculated. Contaminating reads were removed using the SCRuB package v. 0.0.1, which uses the negative and positive control samples to statistically estimate contaminants and remove them [29]. The Phyloseq package v. 1.38.0 [30] was used for further analysis. All figures were made using the ggplot2 package [31] in R.

Bacterial community structure was primarily analysed using variance-stabilizing transformations on the ASV read count matrix data (DESeq2 [32] v. 1.34.0). Variance-stabilizing transformations normalize the count data and stabilize the variance, after which Euclidean distance calculations can be applied to measure community dissimilarity between samples [33]. Secondly, to test if the large read depth disparities created by the different sequencing platforms affected community structure, we also rarefied the ASV read count matrix to 10,000 reads using the rarefy_even_depth function in the Phyloseq [30] package. Following standard practice after rarefaction [34], a Hellinger transformation was applied to the rarefied ASV matrix to normalize the data and reduce the influence of highly abundant taxa. Bray–Curtis distances were calculated using the Vegan package (v. 2.6.4) [35] to measure community dissimilarity. Community structure was visualized through principal coordinate analysis (PCoA) of the non-rarefied and rarefied count data. To determine significant factors affecting bacterial community structure, PERMANOVA (permutational multivariate analasys of variance) was performed on the distance matrixes with treatment and total sequencing reads as interaction terms in the model (Vegan [35]).

Beta diversity dispersions of both variance-stabilized and Hellinger-transformed distance matrices were measured (Vegan [35]) to understand the homogeneity of dispersions based on sequencing platform and library preparation method. A Tukey HSD test was performed to determine differences in beta diversity dispersions between treatments. Additionally, to better identify differences between treatments resulting from the PERMANOVA, a comparison of beta diversity dissimilarity means both within and between treatments was performed using the phyloseq_group_dissimilarity function from the metagMisc package (v. 0.5.0) [36]. An ANOVA was performed on the dissimilarity values to determine the effect of sequencing platform and library preparation method, followed by a Tukey HSD post hoc test to identify differences in treatment dissimilarity means. In all instances of Tukey’s HSD test, *P*-values were adjusted using the Bonferroni correction.

Alpha diversity was compared between automated and manual library preparation methods sequenced on the Illumina MiSeq. The Shannon diversity was calculated from the ASV read count matrix (Vegan [35]) and then correlated using a linear regression model to determine their similarities.

### Positive control analysis

Additional positive control samples were prepared to compare three library preparation methods with a standard mock community. Library preparation was performed manually with manual bead cleanups (Man1) and manually with Opentrons OT-2 bead cleanups (Man2) and automated with KingFisher bead cleanups (Auto1). Each preparation method included five replications and one negative control. Libraries were sequenced using the NextSeq 1000 with a P1 XLEAP-SBS cartridge and a 40% PhiX spike-in. Sequences were processed using DADA2 as previously described. Relative abundances were calculated from the ASV read count matrix (Vegan [35]) and visualized using stacked bar charts. The ASVs belonging to genera in the positive control were plotted, with all others grouped as ‘Other.’ The SCRuB package [29] was used to estimate contamination levels.

## Results

### Design of a miniaturized, automated method for library preparation

We developed an automated protocol for two-step amplicon library preparation utilizing an acoustic liquid handler and an automated magnetic bead cleanup protocol. The acoustic liquid handler was incorporated into both PCR steps as well as library quantification. In both PCR stages, the PCR reaction volume was reduced fivefold (from 25 to 5 µl and from 50 to 10 µl, respectively). We also developed a protocol for automated magnetic bead cleanup using the KingFisher Apex to automate the small reaction volume bead cleanup after each PCR step (Fig. S2). The differences in steps between the automated and manual methods were due to the miniaturization of reaction volumes. Manual gel electrophoresis was replaced with the TapeStation (an automated electrophoresis machine) that accommodates small input DNA volumes. Lastly, the library quantification step was also miniaturized from 200 µl (PicoGreen) to 62 µl (Qubit) with the acoustic liquid handler to limit the library input volumes.

### Comparison of sample data

The total raw read count of the automated method (4,499,351) was higher than that of the manual method (3,905,945) for samples sequenced on the Illumina MiSeq (Fig. S3). The average sample depth was 46,868 and 40,687 reads for the automated and manual methods, respectively. The sample depth ranged from 107 to 128,360 for the automated method and 2,700 to 104,444 for the manual method before filtering. After all filtering steps, there was a total of 3,456,218 reads for the automated method and a total of 3,059,429 reads for the manual method, indicating that 76.82% and 78.33% of reads were retained, respectively. The raw read count for the NextSeq was 12,656,175, with an average read depth of 131,835 and a range of 1,089 to 345,522 reads. After all filtering steps, the total read count was 9,867,362, indicating that 77.96% of reads were retained.

**Fig. 2.**
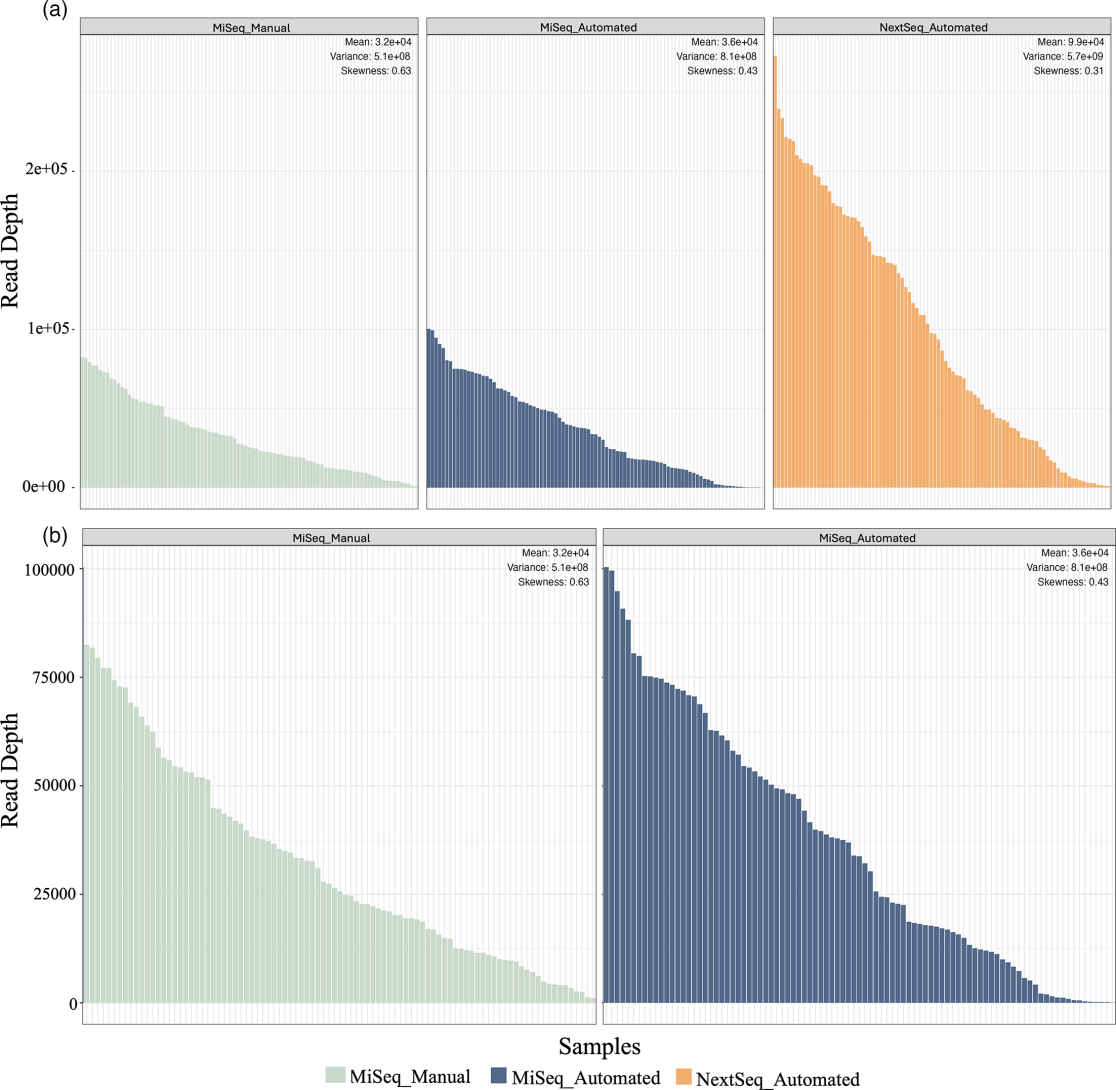
Automated library preparation has generally led to an increase in read depth. (**a**) The distribution of each sample’s read depth across sequencing platform and library preparation method is shown in the above histogram plots. (**b**) The distribution of read depth of samples sequenced on the Illumina MiSeq only. The mean, variance and skewness of each were calculated. Values for these are given in the top right of each figure.

Samples sequenced using either library preparation method with the Illumina MiSeq had more similar read depth distributions when compared with those sequenced with the Illumina NextSeq – suggesting that sequencing technology had a greater effect on read depth skewness between samples (Fig. 2a). However, this could also be due to individual library preparation, sample pooling and library quantification. When comparing the samples sequenced by the Illumina MiSeq, those prepared with the automated method generally had a higher read depth than those prepared with the manual method (Fig. 2b). However, this could be due to the final concentration of pooled libraries and human errors associated with manual pooling and loading of the sequencing cartridge. Samples prepared manually had a lower read depth variance (5.1×108^8^) but a higher level of skewness (0.63), whereas samples prepared with the automated method had a higher read depth variance (8.1×108^8^) but a lower skewness (0.43; Fig. 2b).

**Fig. 3.**
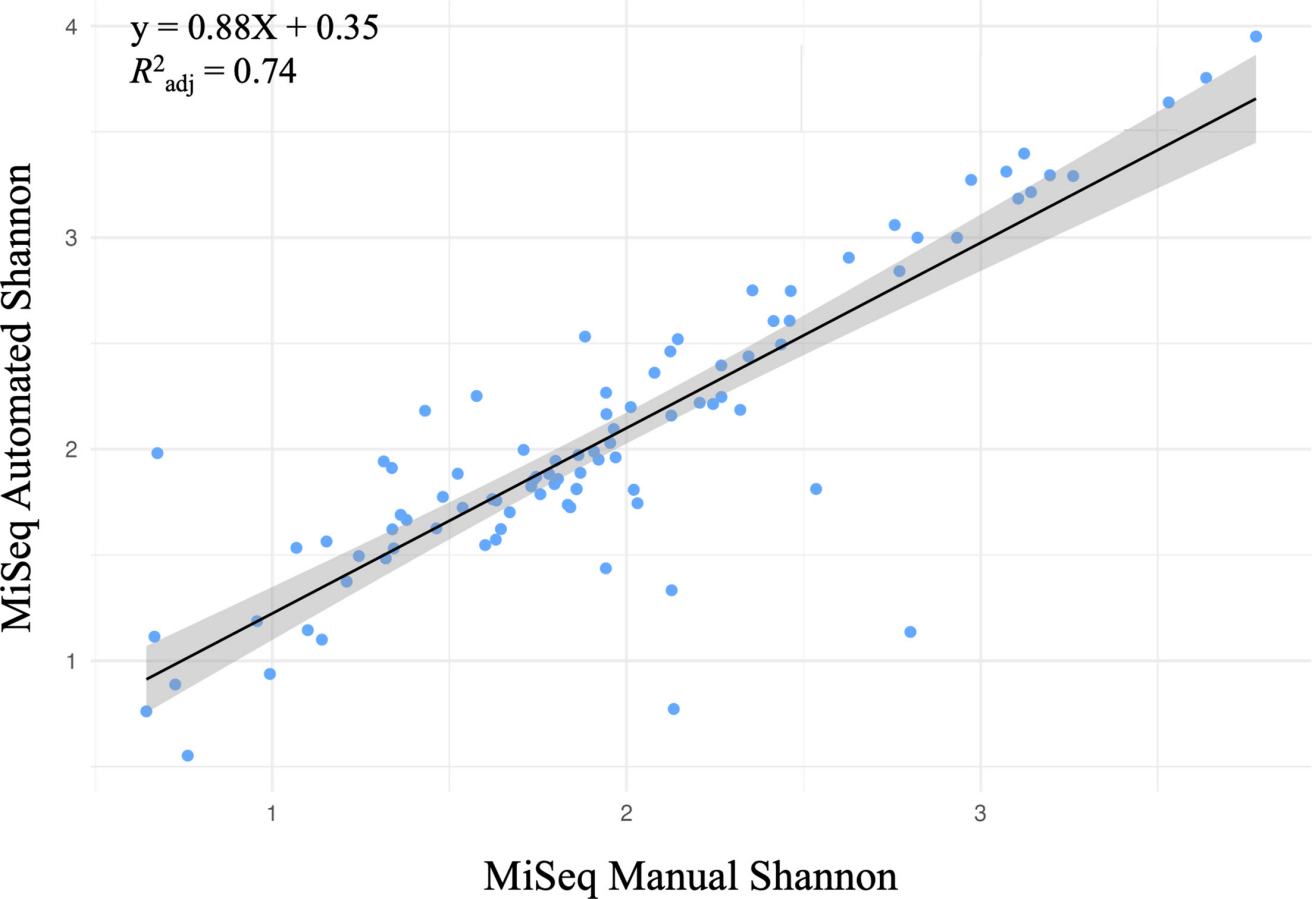
Library preparation methods have similar bacterial alpha diversity metrics. Correlation of Shannon alpha diversity values of automated and manual library preparation methods sequenced on the Illumina MiSeq. Data points represent individual samples.

The Shannon diversity of samples was highly correlated (*R*^2^_adj_ 0.74; Fig. 3), indicating the comparability of the manual and automated library preparation methods in taxa detection.

**Fig. 4.**
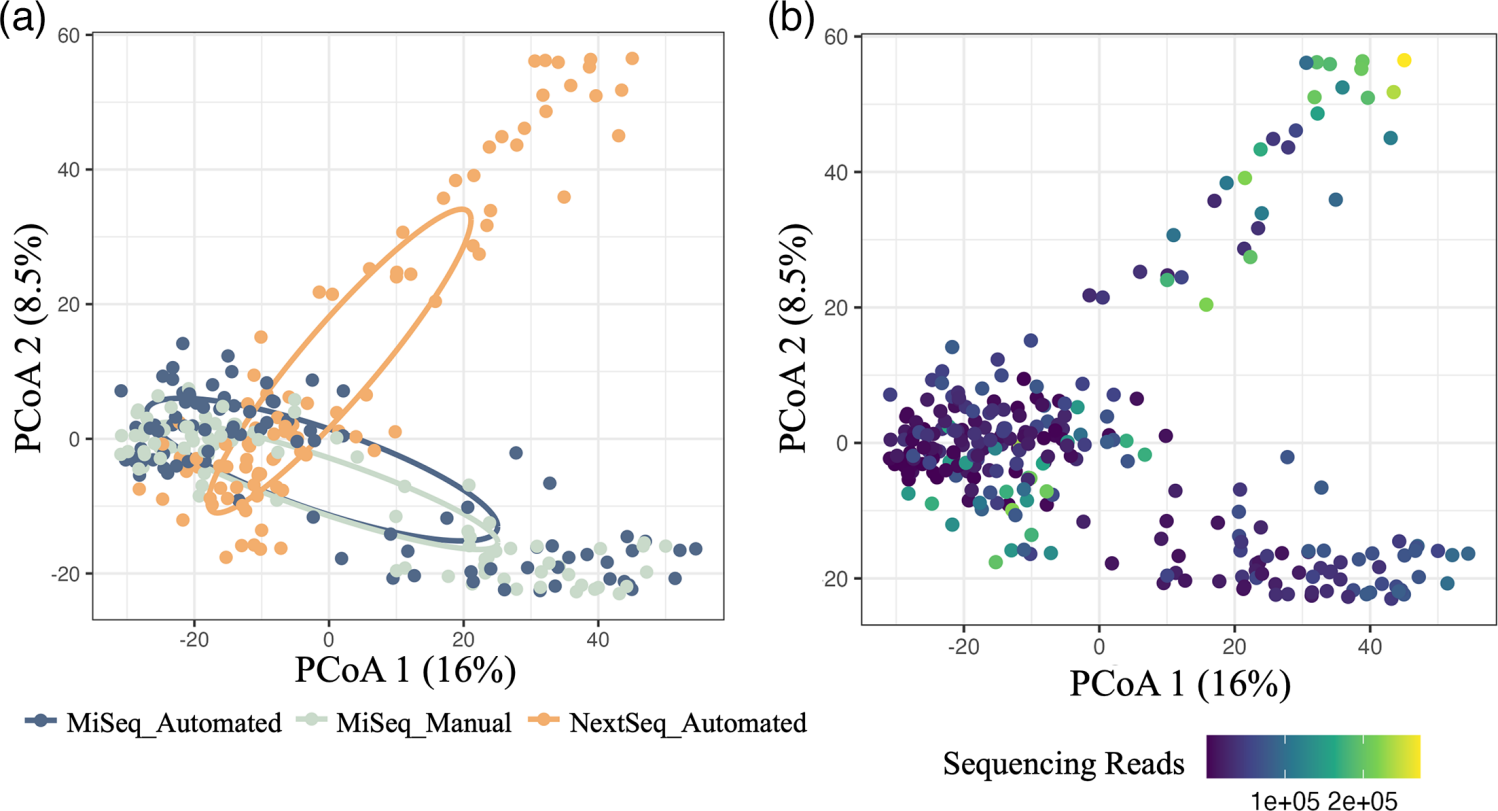
Bacterial community structure varies by sequencing platform but not library preparation method, due to sequencing depth differences. PCoA plots showing community structure differences of variance-stabilized data with Euclidean distances by (a) sequencing platform and library preparation method and (b) sequencing depth. A lighter colour represents a greater number of reads. Each point represents a single sample. *P*-values: sequencing platform and library preparation method × read depth interaction (0.001***), sequencing platform and library preparation method (0.001***) and read depth (0.001***).

Bacterial community structure varied by sequencing platform depending on library preparation method (interaction *P*-value 0.001***; Fig. 4a). Additionally, bacterial community structure also differed by sample read depth depending on sequencing platform and library preparation method (interaction *P*-value 0.001***). Samples sequenced on the Illumina NextSeq showed a greater beta-dispersion in community composition compared with both MiSeq preparation methods (all *P*-value<0.001***; Fig. S5A), which was likely a result of the increased read depth compared with the MiSeq (Fig. 4b). Additionally, samples sequenced on the NextSeq had higher average beta diversity dissimilarities alone and compared with both MiSeq methods. The average dissimilarities for the automated MiSeq method were also higher than the manual MiSeq method. However, the comparison between both MiSeq methods was not different from the within-treatment comparison of both methods (Fig. S6A).

**Fig. 5.**
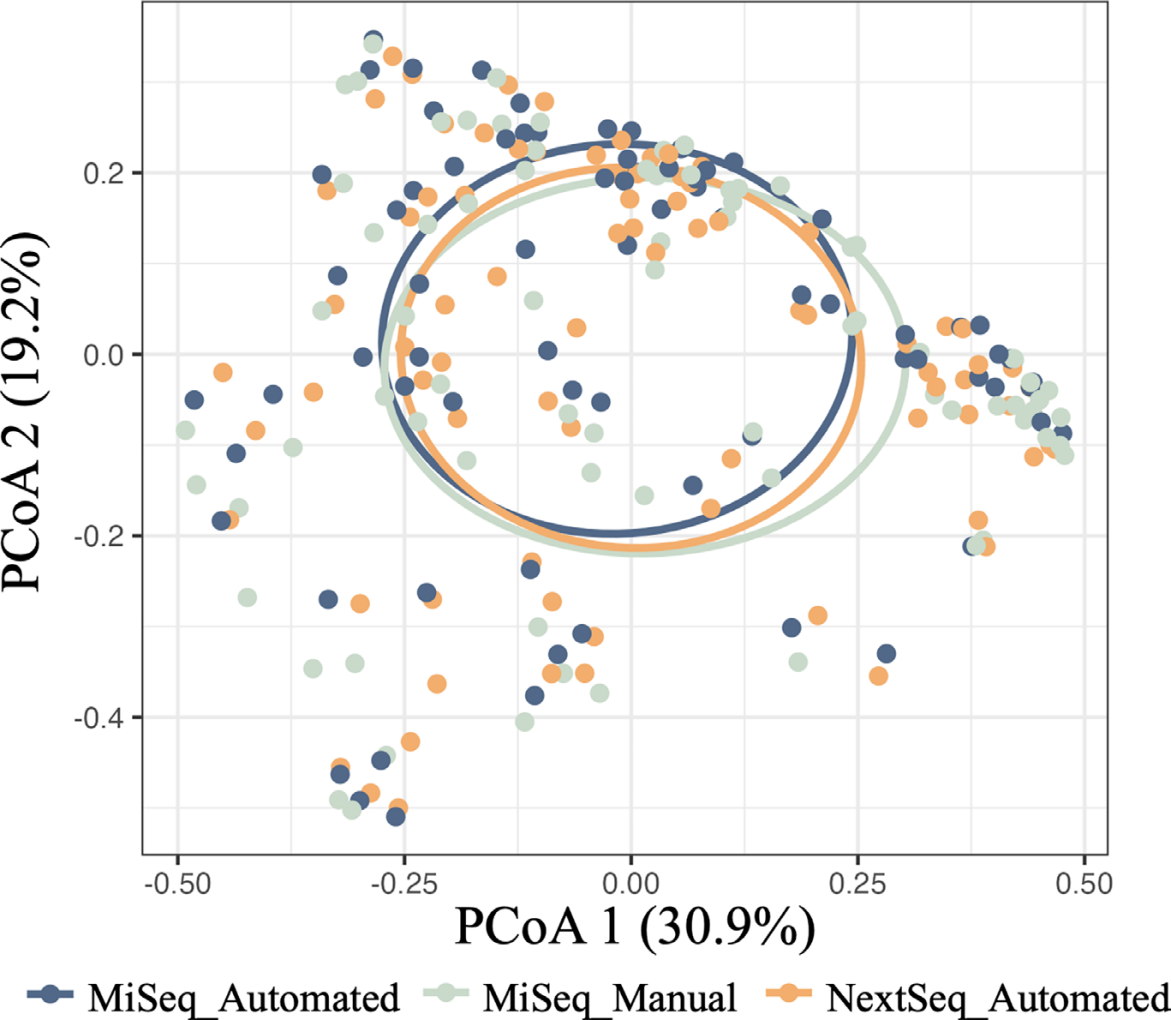
Rarefaction of sequencing results nearly eliminates the differences in community structure due to greater sequencing depth. PCoA plot of sequencing results after rarefying each sample to 10,000 reads. *P*-value: sequencing platform and library preparation method (0.001***).

Rarefaction curves were generated (Fig. S4) and determined that 10,000 reads were the required per sample to capture the majority of taxa present while limiting the number of samples removed due to low read depth. Although samples visually appeared to have small differences in communities between samples sequenced on the MiSeq and NextSeq after rarefaction, there were still significant differences in communities depending on the sequencing platform and library preparation method (*P*-value: 0.001***; Fig. 5). However, beta diversity dispersion was not significantly different between any of the sequencing platforms and library preparation methods (TukeyHSD *P*-adjusted values range from 0.873 to 0.993; Fig. S5B). A comparison of the average beta diversity dissimilarities revealed that while rarefaction has removed the effect of library preparation method for samples sequenced on the MiSeq, it did not completely remove the NextSeq platform effect on community composition (Fig. S6B).

Standardized samples were used to compare the impacts of different library preparation methods on community composition. In this test, we compared automated (Auto1) and manual (Man2) methods described above and added a further manual comparison that included by-hand bead cleanup (Man1). Relative abundance analysis showed that all members present in the standard samples were present at similar compositions across each of the different library preparation methods (Fig. S7). We found that the by-hand bead cleanup method (Man1) had the highest level of contamination in negative control samples (14.08% estimated by SCRuB), with Man2 and Auto1 methods having similar levels of contamination (0.48% and 0.50%, respectively). Overall, these results support that the automated method has not impacted the ability to profile bacterial communities.

### Comparison of quantification

**Fig. 6.**
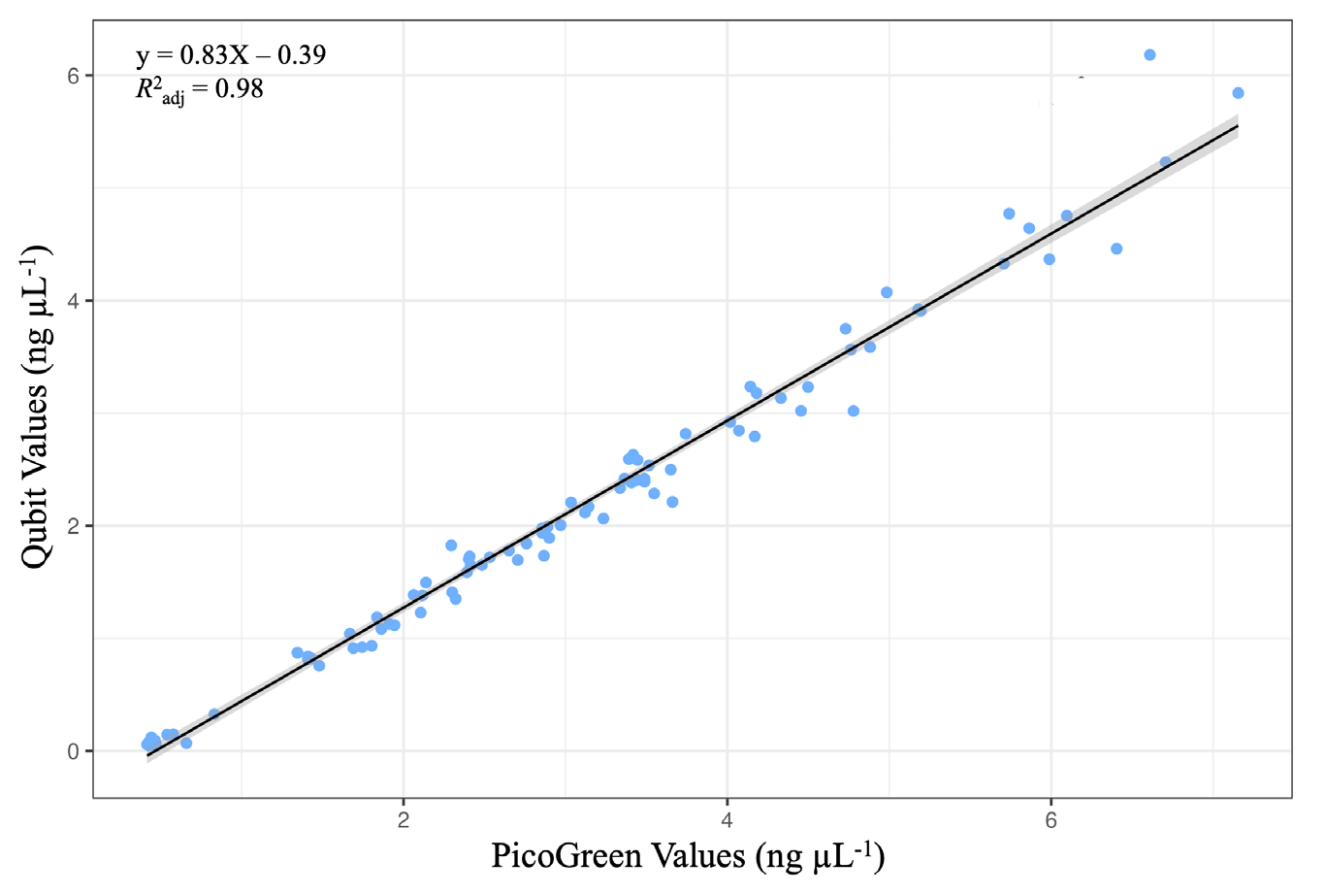
Manual PicoGreen and automated Qubit quantification methods are strongly correlated. Correlation of DNA quantification with PicoGreen (manual) and Qubit (automated). Data points represent individual samples. Quantification measured in nanograms per microlitre. The line represents a linear regression model.

After preparation, the PicoGreen and Qubit DNA quantification methods were compared between manual and automated library preparation methods, respectively. Similar to a previous test comparing both quantification methods [37], the automated Qubit quantification method was highly correlated with the manual PicoGreen method (Adj. *R*^2^=0.98; Fig. 6). However, evaluation of the slope from the regression (0.83) indicated that the automated Qubit method tended to have lower sample concentrations, relative to the manual PicoGreen method – though the between-sample comparisons within a method did not vary along the range of concentrations tested.

### Comparison of consumable usage

**Fig. 7.**
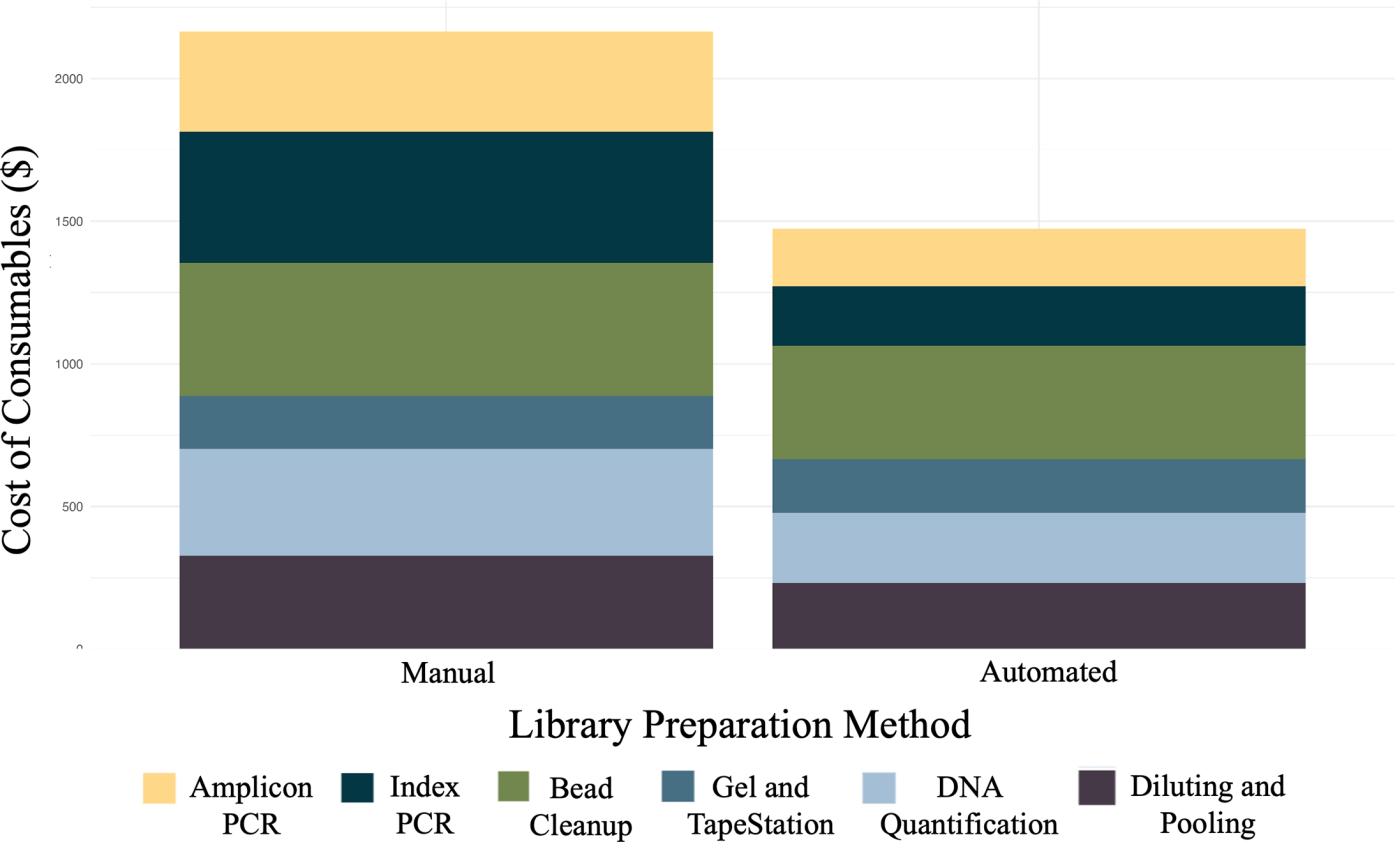
The automated method has decreased the cost of library preparation. The cost of consumables and reagents for each method, coloured by the totals for the library preparation step.

The consumables were tracked for manual and automated library preparation, and the person-hours for each method were also estimated and are presented in Table 1. The development of the automated library preparation protocol led to a reduction in costs as well as person-hours (Table 1). The price reductions for each step in the library preparation were directly compared. There was a ~50% reduction in price for both the amplicon and index PCRs using the automated method (Fig. 7). There was a ~15–30% reduction in costs for all other steps, except for gel electrophoresis and TapeStation sample check steps – where the automated and manual method costs were comparable (Fig. 7). The cost to prepare libraries for one 96-well plate of samples using the manual method was ~$2,200, while the automated method was reduced to ~$1,500 (Table 1), which led to a ~30% reduction in total costs. The total costs of consumables were tracked and totalled for each step and each method (Table S1). There was a ~50% reduction in person-hours to prepare libraries with the automated method (6.5 h) compared with the manual method (12.42 h). Thus, the automated library preparation protocol decreased both the costs and time to prepare libraries.

**Table 1.** The automated library preparation method has decreased the time and cost of library preparation compared with the manual method.Table comparing consumables and reagents used for both manual and automated library preparation. Volumes or numbers of items used are totalled for each reagent or consumable. Prices for each consumable are given per preparation method. Estimated person-hour totals are given per preparation method. NA indicates supplies were not used for the corresponding method

Consumable	Manual amount	Price for manual ($)	Automated amount	Price for automated ($)
2 µM V4_515F primer	250 µl	0.45	75 µl	0.14
2 µM V4R primer	250 µl	0.47	75 µl	0.14
1 µM F Index primers	60 µl	0.11	35 µl	0.06
1 µM R Index primers	40 µl	0.06	35 µl	0.06
20 µM mPNA	250 µl	55	na	na
20 µM pPNA	250 µl	55	na	na
50 µM mPNA	na	na	30 µl	16.5
50 µM pPNA	na	na	30 µl	16.5
KAPA HiFi HotStart ReadyMix	3,750 µl	321.48	1,125 µl	96.44
Nuclease-free water	21 ml	2.86	5,240 µl	0.71
Agarose	4.2 g	1.44	na	na
0.5X TAE	300 ml	1.42	na	na
MIDORI Green Advance DNA Stain	18 µl	2.02	na	na
1 kb ladder	7.5 µl	0.52	na	na
TapeStation buffer	12 µl	2.56	46 µl	9.8
TapeStation ladder	1 µl	7.18	4 µl	28.7
80% (manual) or 70% (automated) ethanol	100 ml	15.35	20 ml	3.03
Magnetic beads	7,296 µl	97.96	1,840 µl	24.7
1X TE buffer	24.0145 ml	15.93	na	na
PicoGreen Quant-iT reagent	62.5 µl	37.38	na	na
2 µg ml^−1^ Lambda DNA	220 µl	1.35	na	na
Qubit 1X dsDNA working solution buffer	na	na	7,276 µl	11.93
Qubit 0 ng µl^−1^ standard	10 µl	na	30 µl	na
Qubit 10 ng µl^−1^ standard	10 µl	na	30 µl	na
Qubit dsDNA HS buffer	995 µl	na	na	na
Qubit reagent	5 µl	2.62	na	na
P20 tips	7–8 boxes+78 tips	852.03–961.09	5–6 boxes+68 tips	622.55–731.61
P200 tips	4 boxes+57 tips	500.99	3 boxes+78 tips	415.79
P1000 tips	17 tips	19.31	1 tip	1.14
OT-2 P200 Tips	12.66 boxes	100.01	na	na
384-well source plate	na	na	1 plate	10.27
384-well destination plate	na	na	1 plate	13.76
96-well PCR plates – Thermo Armadillo High Performance	na	na	10 plates	51.7
Black-edged 96-well plates	1 plate+23 wells	12.64	na	na
Black-edged 384-well plates	na	na	98 wells	3.66
12-well deep well plate	2	21.74	na	na
1.7 ml microfuge tube	1 tube	0.03	1 tube	0.03
Qubit tubes	5 tubes	1.15	4 tubes	0.92
PCR strip tubes	5 tubes	2.45	31 tubes	15.19
TapeStation tips	5 tips	4.06	23 tips	18.69
High Sensitivity D1000 ScreenTape TapeStation Lanes	5 lanes	18.35	23 lanes	84.41
Tip comb	na	na	2	17.56
Trough	2	2.04	3	3.06
Plate seals	9	7.92	7	6.16
**Total price**		2,162.53–2,271.59		1,473.60–1,582.66
**Estimated total person-hours**		12.42		6.5

## Discussion

The availability and reduced costs of sequencing have led to an increase in studies utilizing high-throughput sequencing technologies. However, there is still a significant expense for the preparation of samples for sequencing due to reagent and consumable costs. The incorporation of an acoustic liquid handler enabled the development of a library preparation protocol that decreased the preparation time and reaction volumes, ultimately increasing the efficiency of material use and lowering user expenses (Fig. 1, Table 1).

We compared this automated method to the standard, manual preparation. The automated library preparation method led to a higher average read depth and a more symmetrical distribution in sequencing (Fig. 2). The taxa detection and Shannon diversity of both automated and manual methods were comparable (Fig. 3). When analysing the bacterial community compositions, we saw that while library preparation method significantly affected these communities, a majority of this effect was due to an increase in sequencing read depth (Fig. 4). After rarefaction, this effect was still significant but much lower, and there were fewer differences in community compositions (Figs 5 and S6). This could be due to the increase in read depth seen with the automated library preparation method, which would allow for a greater understanding of microbial communities including rare or low-abundance taxa [38,39]. A comparison of standardized samples including automated and two manual methods (incorporating Opentrons OT-2 or by-hand bead cleanups) also demonstrated a similar measurement of compositions in positive control samples (Fig. S7). The utilization of automated bead cleanups may have also reduced contamination observed in negative control samples, whether utilizing the Opentrons OT-2 robot or a KingFisher Apex.

The automation and miniaturization reduced the time (~50% reduction) and cost (~30% reduction) of preparing samples for amplicon sequencing (Fig. 7, Table 1). The manual method in this study primarily utilized an Opentrons OT-2 robot to perform bead cleanup; however, all steps and reagent volumes were the same between the by-hand and by-robot bead cleanup procedures. Incorporating the OT-2 robot further reduced the times of bead cleanup in the manual method from ~2.5 h per bead cleanup by hand to ~2 h by robot. An entirely manual method would then take ~13.5 person-hours to complete. Our cost of preparation for one 96-well plate was over $2,000 for the manual method or ~$23 per sample. The automated method has reduced that cost to around $1,500 or ~$16 per sample. This approach not only lowers expenses but also supports the environmental sustainability of research by limiting plastic waste.

There are several studies using acoustic liquid handlers to miniaturize and automate different types of molecular and cellular assays [15,20], as well as one for amplicon sequencing [21]. In this previous report, EMP researchers miniaturized a one-step library preparation method using an acoustic liquid handler and compared it to a pipette-based liquid handler, which led to a ~58% reduction in library preparation costs. Our method led to a ~30% reduction in expenses due to miniaturization. However, this is likely because we used a two-step PCR process, which requires more polymerase, magnetic beads and transfers between PCRs and bead cleanups compared with the one-step method. The two-step PCR method has several advantages, including cheaper primers, reduced amplification bias when compared with the long adapter index primers required in the one-step method [23] and the option to increase sample multiplexing by introducing a second layer of barcodes during amplicon PCR [24]. While the previous group successfully miniaturized an amplicon library preparation protocol using an acoustic liquid handler, we combined an acoustic liquid handler and an automated magnetic bead cleanup system for a two-step PCR library preparation process.

We successfully developed a method that miniaturized and reduced the costs of amplicon library preparation for microbiome studies compared with the standard, manual method. While this method was tested on the V4 region of the 16S rRNA gene, other regions of the 16S rRNA gene and the ITS region could be sequenced using the corresponding primer sets. Our automated protocol is available for further optimization and cost-saving changes. For example, since the initial test of this protocol, we have reduced tip waste by eliminating a sample transfer between 384-well and 96-well plates after PCR amplification prior to bead cleanup. This was accomplished by simply using 96-well plates throughout all steps and further reduced sample costs from $16 to $13 per sample, which will almost fully offset the costs of a sequencing cartridge. However, since the cost of an acoustic liquid handler can range between $200,000 to $500,000, even the optimized automated method would require ~208 to 521 96-well plates to be processed to offset the cost of purchase. Thus, a key limitation of this method is the necessity to use specific equipment, including the acoustic liquid handler and automated magnetic bead cleanup robot, which may only be practical if such equipment is already available.

## Conclusions

Due to the need to reduce the bottleneck of library preparation in amplicon sequencing, we successfully developed a miniaturized and automated method utilizing an acoustic liquid handler paired with automated magnetic bead cleanup. The ability of the liquid handler to transfer small volumes of liquid increased efficiency in consumables and reagent usage and reduced time for completion compared with the manual method. A major benefit of the automated preparation method is the reduction in consumable costs and person-hours (Fig. 7, Table 1) with no differences in bacterial communities from the MiSeq (Figs 5 and S5). The increased consumable efficiency and reduced time to prepare libraries using this method will lead to significant cost savings to prepare hundreds of samples in future microbiome studies.

## Supplementary material

10.1099/mic.0.001579 Uncited Supplementary Material 1.

## Abbreviations

ASV: amplicon sequencing variant
EMP: Earth Microbiome Project
FHB: *Fusarium* head blight
NGS: next-generation sequencing
PCoA: principal coordinate analysis
PERMANOVA: permutational multivariate analasys of variance
PNA: peptide nucleic acid
