# Supplementary material for: Improved efficiency of two-step amplicon PCR using an acoustic liquid handler
Source: Microbiology (Reading). 2025 Jul 22;171(7):001579. doi: 10.1099/mic.0.001579 (PMC12283028; doi:10.1099/mic.0.001579)
Supplement: Uncited Supplementary Material 1. [file mic-171-01579-s001.pdf]

Supplementary Information

A)

Genotypes Used
Klages
2Ab09-X06M035-45
2Ab09-X05M056-15
2Ab10-X08M260-40
10ARS022-2
11ARS148-4
13ARS079-3
11S145-9
12ARS039-2
2Ab11-X08M256-10

B)

Location	Longitude	Latitude	Climate
Kimberly, ID	42.533169	-114.364022	Semi-arid
St. Paul, MN	44.949642	-93.093124	Humid continental
Fargo, ND	46.877186	-96.789803	Continental
Ithaca, NY	42.443962	-76.501884	Warm-summer humid continental

Supplementary Figure 1. A) 10 specific barley genotypes from the spring malt barley training population. **B)** Longitude, latitude, and climate type of the four FHB-misted nursery locations.

Step	Description
1	Pick up 96 PCR tip comb
**2**	Bind to magnetic beads in each sample
**3**	Wash 1 with 70% EtOH
**4**	Wash 2 with 70% EtOH
**5**	Collect beads
**6**	Dry beads
**7**	Elute
**8**	Collect beads
**9**	Collect beads
**10**	Leave 96 PCR tip comb

Supplementary Figure 2. Steps for small volume magnetic bead cleanup program for the KingFisher Apex.

MiSeq Manual									
**Stat**	**rawreads**	**filtered**	**denoisedF**	**denoisedR**	**merged**	**nonchim**	**nochloro**	**nomito**	**noeuk**
**TotalReadCount**	3905945	3634279	3586780	3586704	3519625	3473076	3438820	3094694	3059429
**AverageReadCount**	40686.9271	37857.0729	37362.2917	37361.5	36662.7604	36177.875	35821.0417	32236.3958	31869.0521
**RangeReadCount**	101744	95765	95048	95180	93759	92774	92476	83561	82868
**PercentReadsRemaining**		93.0448073	91.828738	91.8267922	90.1094357	88.9176883	88.0406662	79.2303527	78.3274982
**MiSeq Automated**									
**Stat**	**rawreads**	**filtered**	**denoisedF**	**denoisedR**	**merged**	**nonchim**	**nochloro**	**nomito**	**noeuk**
**TotalReadCount**	4499351	4216702	4179074	4179727	4088519	3925108	3905869	3483466	3456218
**AverageReadCount**	46868.2396	43923.9792	43532.0208	43538.8229	42588.7396	40886.5417	40686.1354	36286.1042	36002.2708
**RangeReadCount**	128253	120439	119666	119726	118186	117131	116801	103585	101111
**PercentReadsRemaining**		93.7180051	92.8817067	92.8962199	90.8690831	87.2372038	86.8096088	77.4215215	76.815923
**NextSeq Automated**									
**Stat**	**rawreads**	**filtered**	**denoisedF**	**denoisedR**	**merged**	**nonchim**	**nochloro**	**nomito**	**noeuk**
**TotalReadCount**	12656175	11986721	11904587	11913387	11651142	10988473	10929990	9941576	9867362
**AverageReadCount**	131835.156	124861.677	124097.781	124097.781	121366.063	114463.26	113854.063	103558.083	102785.021
**RangeReadCount**	344433	328157	325503	325466	317030	307283	306979	287359	285519
**PercentReadsRemaining**		94.7104556	94.0614917	94.131023	92.0589515	86.8230172	86.3609266	78.5511894	77.9648037

Supplementary Figure 3. Tracking the read count, average read counts, range of read counts, and percent of reads remaining after each filtering step in DADA2.

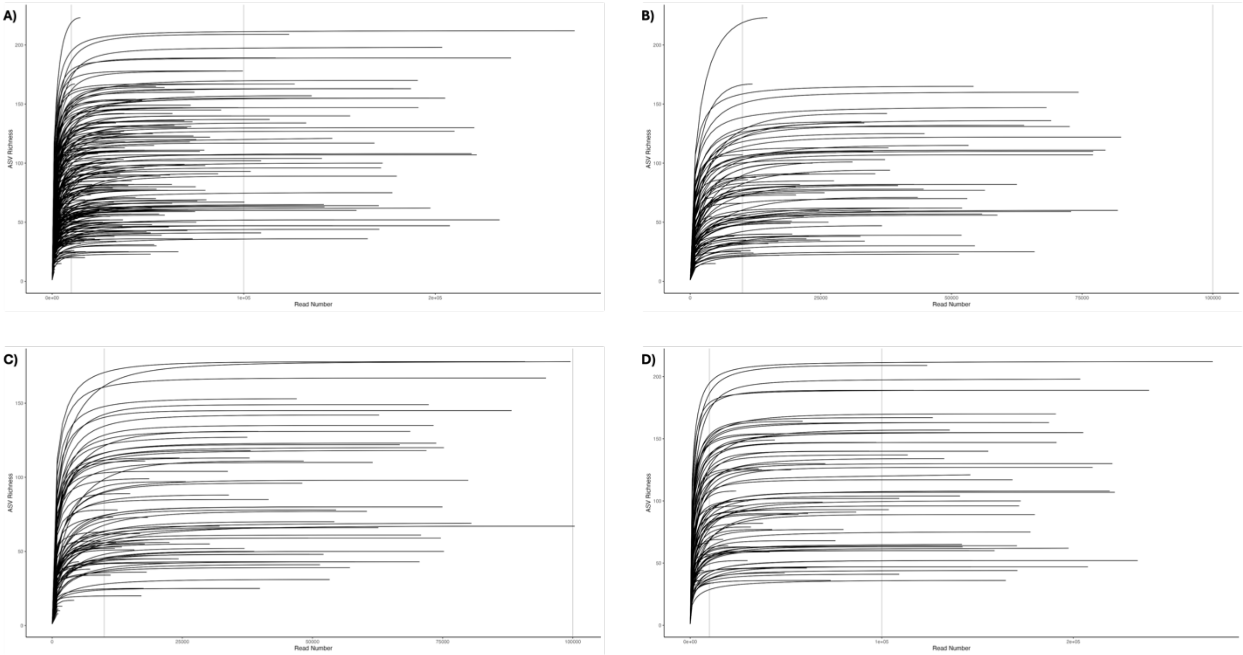

Supplementary Figure 4. Rarefaction curves of each sample showing read depth along the x-axis and ASV richness along the y-axis. The 10,000 and 100,000 read-depths are denoted with vertical grey lines. **A)** Rarefaction curves of all samples across each sequencing platform and library preparation method. **B)** Rarefaction curves of samples prepared manually and sequenced by the MiSeq. **C)** Rarefaction curves of samples prepared with the acoustic liquid handler and sequenced by the MiSeq. **D)** Rarefaction curves of samples prepared with the acoustic liquid handler and sequenced by the NextSeq.

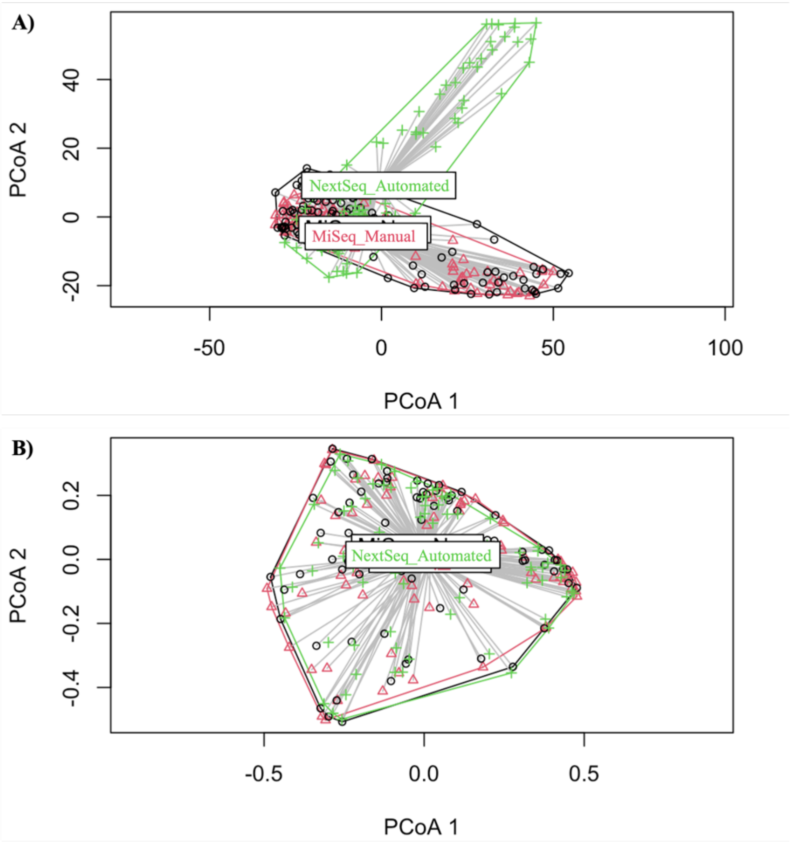

Supplementary Figure 5. Beta diversity dispersion of sequencing platform and library preparation methods. Each point represents a single sample. Significance was tested using a Tukey HSD test. **A)** Dispersion of samples before rarefaction. P-adjusted values: MiSeq Manual x MiSeq Automated (0.217), NextSeq Automated x MiSeq Manual (<0.001***), NextSeq Automated x MiSeq Automated (<0.001***). **B)** Dispersion of samples after rarefaction. P-adjusted values: MiSeq Manual x MiSeq Automated (0.873), NextSeq Automated x MiSeq Manual (0.919), NextSeq Automated x MiSeq Automated (0.993).

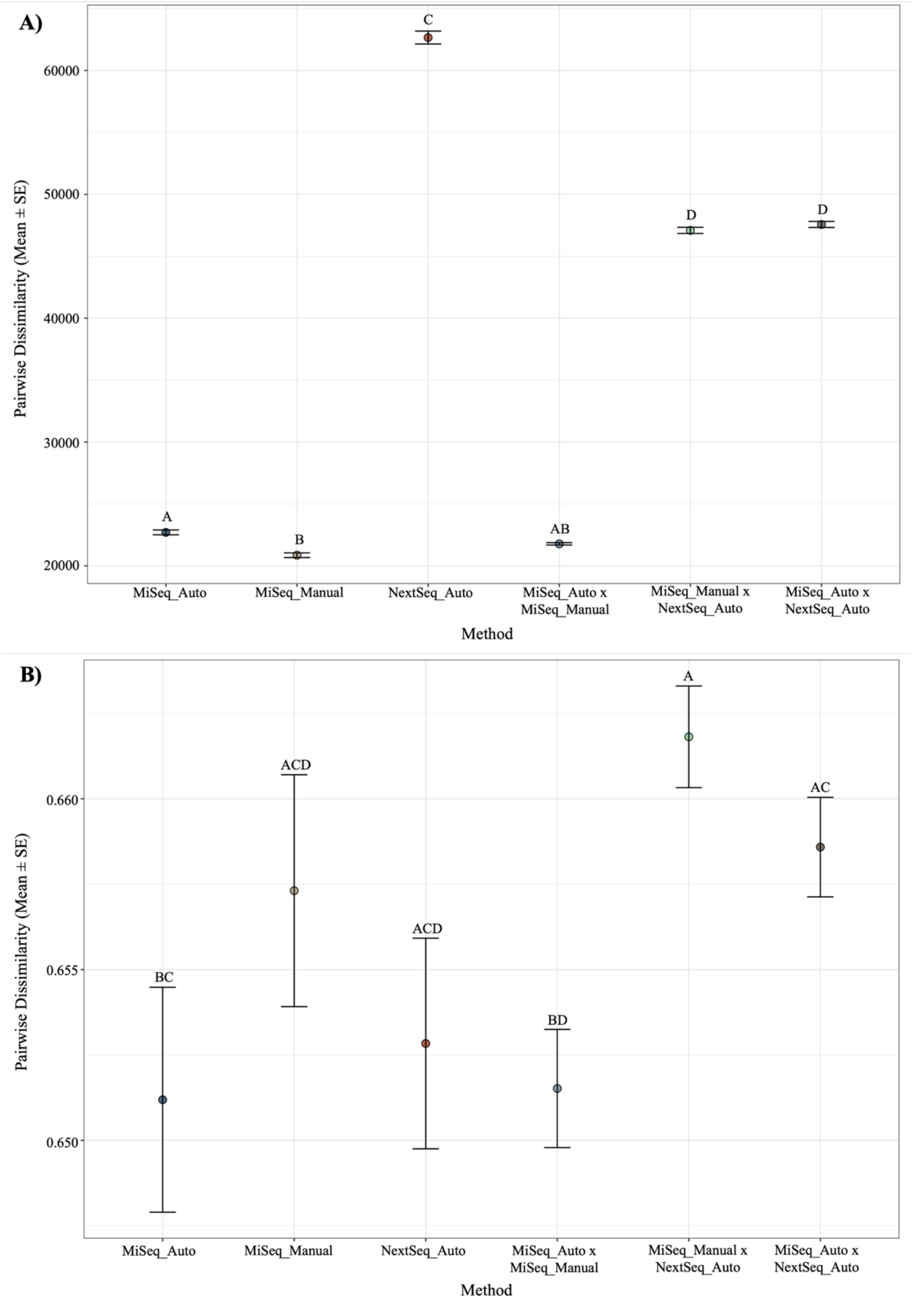

Supplementary Figure 6. Comparison of beta diversity dissimilarity means for each sequencing platform and library preparation method and their pairwise comparisons. Significance was determined with a Tukey HSD test. **A)** Comparison of means before rarefaction using Euclidean distances. **B)** Comparison of means after rarefaction using Bray-Curtis distances.

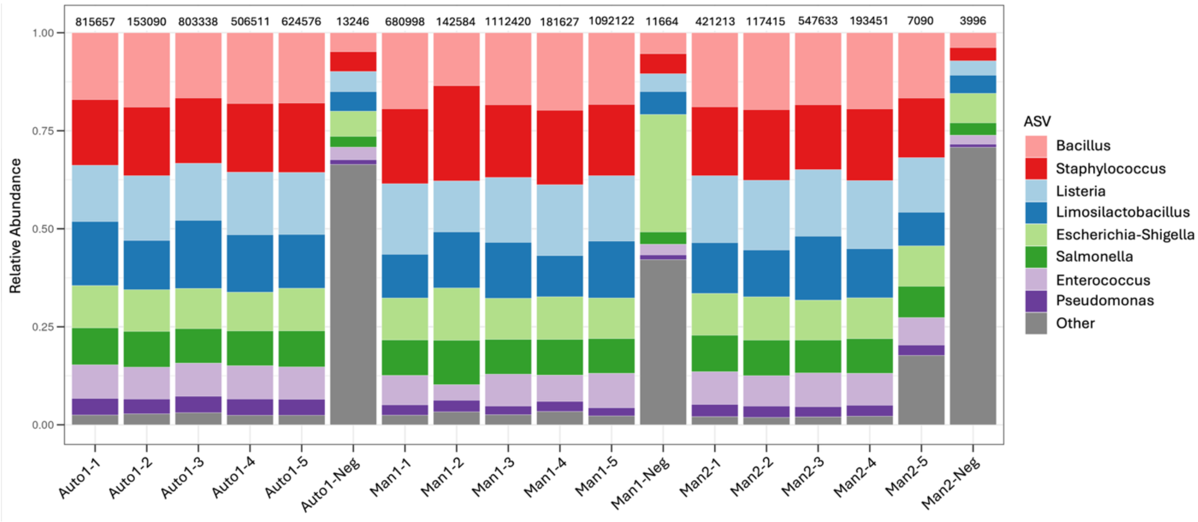

Supplementary Figure 7. Relative abundances of bacterial ASVs colored by genera present in the positive control with all other minor and non-positive control ASVs grouped as "Other". The read depth is given above the bar of each sample.

Library Prep Step	Consumable	Manual	Price for Manual ($)	Automated	Price for Automated ($)
**PCR1**					
	2 uM V4_515F primer	250 µL	0.45	75 µL	0.14
	2 uM V4R primer	250 µL	0.47	75 µL	0.14
	20 uM mPNA	250 µL	55	NA	NA
	20 uM pPNA	250 µL	55	NA	NA
	50 uM mPNA	NA	NA	30 µL	16.5
	50 uM pPNA	NA	NA	30 µL	16.5
	KAPA HiFi HotStart ReadyMix	1250 µL	107.16	375 µL	32.15
	Nuclease-Free Water	NA	NA	90 µL	0.01

	Sample DNA	2.5 µL	NA	0.5 µL	NA
	P20 tips	1 box	109.06	1 box	109.06
	P200 tips	15 tips	17.04	7 tips	7.95
	P1000 tips	5 tips	5.68	NA	NA
	384 well Source Plate	NA	NA	1 plate	10.27
	384 well Destination plate	NA	NA	1/2 plate	6.88
	96 well PCR plates - Thermo Armadillo High Performance	NA	NA	NA	NA
	Plate seals	2 seals	1.76	2 seals	1.76
	**Total ($)**		**351.62**		**201.36**
**Electrophoresis Gel**					

	Agarose	1.4 g	0.48	NA	NA
	0.5X TAE	100 mL	0.47	NA	NA
	MIDORI Green Advance DNA Stain	6 μL	0.67	NA	NA
	1 kb Ladder	2.5 μL	0.17	NA	NA
	Sample DNA	3 μL	NA	NA	NA
	P20 tips	41 tips	46.58	NA	NA
	**Total ($)**		**48.37**		**NA**
**Agilent TapeStation**					
	TapeStation Buffer	NA	NA	10 μL	2.13
	Nuclease-Free Water	NA	NA	10 μL	0.001

	TapeStation Ladder	NA	NA	0.5 µL	3.59
	Sample DNA	NA	NA	0.5 µL	NA
	PCR Strip Tubes	NA	NA	5 tubes	2.45
	P20 tips	NA	NA	7 tips	7.95
	TapeStation Tips	NA	NA	5 tips	4.06
	TapeStation Lanes	NA	NA	5 lanes	18.35
	**Total ($)**		**NA**		**38.531**
**Bead Cleanup**					
	80% (manual) or 70% (automated) Ethanol	50 mL	7.67	10 mL	1.51
	Nuclease-Free Water	10 mL	1.36	2560 µL	0.35

	Magnetic Beads	1920 µL	25.78	800 µL	10.75
	OT-2 P200 Tips	6.33 boxes	50.01	NA	NA
	P20 tips	1 box	109.06	1 box + 16 tips	127.24
	P200 tips	NA	NA	23 tips	26.13
	P1000 tips	3 tips	3.41	NA	NA
	Plate seals	2	1.76	1	0.88
	12-well Deep well plate	1	10.87	NA	NA
	96 well PCR plates - Thermo Armadillo High Performance	NA	NA	5 plates	25.85
	Tip Comb	NA	NA	1	8.78
	Trough	NA	NA	1	1.02

	**Total ($)**		**209.92**		**202.51**
**Electrophoresis Gel**					
	Agarose	1.4 g	0.48	NA	NA
	0.5X TAE	100 mL	0.47	NA	NA
	MIDORI Green Advance DNA Stain	6 μL	0.67	NA	NA
	1 kb Ladder	2.5 μL	0.17	NA	NA
	Sample DNA	3 μL	NA	NA	NA
	P20 tips	41 tips	46.58	NA	NA
	**Total ($)**		**48.37**		**NA**
**Agilent TapeStation**					

	TapeStation Buffer	NA	NA	10 µL	2.13
	Nuclease-Free Water	NA	NA	10 µL	0.001
	TapeStation Ladder	NA	NA	0.5 µL	3.59
	Sample DNA	NA	NA	0.5 µL	NA
	PCR Strip Tubes	NA	NA	5 tubes	2.45
	P20 tips	NA	NA	7 tips	7.95
	TapeStation Tips	NA	NA	5 tips	4.06
	TapeStation Lanes	NA	NA	5 lanes	18.35
	**Total ($)**		**NA**		**38.531**
**PCR2**					

	1 uM F Index primers	60 µL	0.11	35 µL	0.06
	1 uM R Index primers	40 µL	0.06	35 µL	0.06
	Nuclease-Free Water	1000 µL	0.14	150 µL	0.02
	KAPA HiFi HotStart ReadyMix	2500 µL	214.32	750 µL	64.3
	Sample DNA	5 µL	NA	2 µL	NA
	P20 tips	1 box + 19 tips	130.64	1 box	109.06
	P200 tips	1 box	109.06	22 tips	24.99
	P1000 tips	3 tips	3.41	1 tip	1.14
	384 well Destination plate	NA	NA	1/2 plate	6.88

	96 well PCR plates - Thermo Armadillo High Performance	NA	NA	NA	NA
	Plate seals	2	1.76	2	1.76
	**Total ($)**		**459.5**		**208.27**
**Electrophoresis Gel**					
	Agarose	1.4 g	0.48	NA	NA
	0.5X TAE	100 mL	0.47	NA	NA
	MIDORI Green Advance DNA Stain	6 µL	0.67	NA	NA
	1 kb Ladder	2.5 µL	0.17	NA	NA
	Sample DNA	3 µL	NA	NA	NA
	P20 tips	41 tips	46.58	NA	NA

	**Total ($)**		**48.37**		**NA**
**Agilent TapeStation**					
	TapeStation Buffer	NA	NA	10 μL	2.13
	Nuclease-Free Water	NA	NA	10 μL	0.001
	TapeStation Ladder	NA	NA	0.5 μL	3.59
	Sample DNA	NA	NA	0.5 μL	NA
	PCR Strip Tubes	NA	NA	5 tubes	2.45
	P20 tips	NA	NA	7 tips	7.95
	TapeStation Tips	NA	NA	5 tips	4.06
	TapeStation Lanes	NA	NA	5 lanes	18.35

	**Total ($)**		**NA**		**38.531**
**Bead Cleanup**					
	80% (manual) or 70% (automated) Ethanol	50 mL	7.67	10 mL	1.51
	Nuclease-Free Water	10 mL	1.36	2400 μL	0.33
	Magnetic Beads	5376 μL	72.18	1040 μL	13.96
	OT-2 P200 Tips	6.33 boxes	50.01	NA	NA
	P20 tips	NA	NA	1 box + 8 tips	118.15
	P200 tips	1 box	109.06	22 tips	24.99
	P1000 tips	3 tips	3.41	NA	NA
	Plate seals	2	1.76	1	0.88
	12-well Deep well plate	1	10.87	NA	NA

	96 well PCR plates - Thermo Armadillo High Performance	NA	NA	5 plates	25.85
	Tip Comb	NA	NA	1	8.78
	Trough	NA	NA	1	1.02
	**Total ($)**		**256.32**		**195.47**
**Agilent TapeStation**					
	TapeStation Buffer	NA	NA	10 μL	2.13
	Nuclease-Free Water	NA	NA	10 μL	0.001
	TapeStation Ladder	NA	NA	0.5 μL	3.59
	Sample DNA	NA	NA	0.5 μL	NA
	PCR Strip Tubes	NA	NA	5 tubes	2.45

	P20 tips	NA	NA	7 tips	7.95
	TapeStation Tips	NA	NA	5 tips	4.06
	TapeStation Lanes	NA	NA	5 lanes	18.35
	**Total ($)**		**NA**		**38.531**
**PicoGreen Quantification**					
	Black-edged 96 well plates	1.08	11.02	NA	NA
	1X TE Buffer	21.535 mL	14.29	NA	NA
	PicoGreen Quantit Reagent	55 μL	32.89	NA	NA
	2ug/mL Lambda DNA	110 μL	1.35	NA	NA
	Sample DNA	1 μL	NA	NA	NA

	P20 tips	1 box + 6 tips	115.88	NA	NA
	P200 tips	1 box + 22 tips	134.05	NA	NA
	P1000 tips	2 tips	2.27	NA	NA
	Trough	2	2.04	NA	NA
	Plate seals	1	0.88	NA	NA
	**Total ($)**		**314.67**		**NA**
**Qubit Quantification**					
	Black-edged 384 well plates	NA	NA	98 wells	3.63
	Qubit 1X dsDNA Working Solution Buffer	NA	NA	6.5 mL	10.66

	Qubit 0 ng/uL standard	NA	NA	20 μL	NA
	Qubit 10 ng/uL standard	NA	NA	20 μL	NA
	Sample DNA	NA	NA	0.6 μL	NA
	P200 tips	NA	NA	2 boxes + 2 tips	220.39
	Trough	NA	NA	1	1.02
	Plate seals	NA	NA	1	0.88
	**Total ($)**		**NA**		**236.58**
**Diluting to 4 nM**					
	Nuclease-Free Water	Individual	NA	Individual	NA
	Sample DNA	Individual	NA	Individual	NA
	P20 tips	1-2 boxes	109.06-218.12	1-2 boxes	109.06-218.12

	P200 tips	~1 box	109.06	~1 box	109.06
	**Total ($)**		**218.12-327.18**		**218.12-327.18**
**Pooling**					
	P20 tips	1 box	109.06	9 tips	10.22
	1.7 mL microfuge tube	1 tube	0.03	1 tube	0.03
	96 well PCR plates - Thermo Armadillo High Performance	NA	NA	NA	NA
	PCR srtip tubes	NA	NA	8 tubes	3.92
	Sample DNA	5 µL	NA	5 µL	NA
	**Total ($)**		**109.09**		**14.17**

**Pooled Quantification**					
**PicoGreen**					
	96-well black well plates	15 wells	1.59	NA	NA
	1X TE Buffer	2479.5 μL	1.65	NA	NA
	PicoGreen Quantit Reagent	7.5 μL	4.49	NA	NA
	2ug/mL Lambda DNA	110 μL	1.35	NA	NA
	Sample DNA	1 μL	NA	NA	NA
	P20 tips	10 tips	11.36	NA	NA
	P200 tips	15 tips	17.04	NA	NA
	**Total ($)**		**37.48**		**NA**

**Qubit**					
	Qubit tubes	5 tubes	1.15	4 tubes	0.92
	Qubit dsDNA HS Buffer	995 μL	NA	NA	NA
	Qubit Reagent	5 μL	2.62	NA	NA
	1X dsDNA Working Solution Buffer	NA	NA	776 μL	1.27
	Sample DNA	2 μL	NA	2 μL	NA
	Qubit 0 ng/uL standard	10 μL	NA	10 μL	NA
	Qubit 10 ng/uL standard	10 μL	NA	10 μL	NA
	P20 tips	11 tips	12.5	4 tips	4.54
	P200 tips	5 tips	5.68	2 tips	2.27

	P1000 tips	1 tip	1.14	NA	NA
	**Total ($)**		**23.09**		**9**
**Agilent TapeStation**					
	TapeStation Buffer	12 μL	2.56	6 μL	1.28
	Nuclease-Free Water	1 μL	<0	2 μL	<0
	TapeStation Ladder	1 μL	7.18	2 μL	14.36
	Sample DNA	1 μL	NA	0.5 μL	NA
	PCR Strip Tubes	5 tubes	2.45	3 tubes	1.47
	P20 tips	5 tips	5.68	3 tips	3.41
	TapeStation Tips	5 tips	4.06	3 tips	2.44

	TapeStation Lanes	5 lanes	18.35	3 lanes	11.01
	**Total ($)**		**40.28**		**33.97**
**Price totals:**			**2165.2-2274.26**		**1473.57-1582.63**

Supplementary Table 1. Table comparing consumables and reagents used for both manual and automated library preparation. for each step. The total price of each step and overall totals are given.

Biosample	Bioproject	Accession	Sample
SAMN44993558	PRJNA1190462	SRR31505633	Amplicon of barley spike: BM-11 Auto Nextseq
SAMN44993558	PRJNA1190462	SRR31505632	Amplicon of barley spike: BM-11n Auto Miseq
SAMN44993558	PRJNA1190462	SRR31505497	Amplicon of barley spike: BM-11o Man Miseq
SAMN44993558	PRJNA1190462	SRR31505638	Amplicon of barley spike: BM-15 Auto Nextseq
SAMN44993558	PRJNA1190462	SRR31505555	Amplicon of barley spike: BM-15n Auto Miseq
SAMN44993558	PRJNA1190462	SRR31505544	Amplicon of barley spike: BM-15o Man Miseq
SAMN44993558	PRJNA1190462	SRR31505485	Amplicon of barley spike: BM-17 Auto Nextseq
SAMN44993558	PRJNA1190462	SRR31505474	Amplicon of barley spike: BM-17n Auto Miseq
SAMN44993558	PRJNA1190462	SRR31505439	Amplicon of barley spike: BM-17o Man Miseq
SAMN44993558	PRJNA1190462	SRR31505428	Amplicon of barley spike: BM-19 Auto Nextseq
SAMN44993558	PRJNA1190462	SRR31505631	Amplicon of barley spike: BM-19n Auto Miseq
SAMN44993558	PRJNA1190462	SRR31505620	Amplicon of barley spike: BM-19o Man Miseq

SAMN44993558	PRJNA1190462	SRR31505609	Amplicon of barley spike: BM-2 Auto Nextseq
SAMN44993558	PRJNA1190462	SRR31505598	Amplicon of barley spike: BM-2n Auto Miseq
SAMN44993558	PRJNA1190462	SRR31505587	Amplicon of barley spike: BM-2o Man Miseq
SAMN44993558	PRJNA1190462	SRR31505528	Amplicon of barley spike: BM-201 Auto Nextseq
SAMN44993558	PRJNA1190462	SRR31505517	Amplicon of barley spike: BM-201n Auto Miseq
SAMN44993558	PRJNA1190462	SRR31505458	Amplicon of barley spike: BM-201o Man Miseq
SAMN44993558	PRJNA1190462	SRR31505447	Amplicon of barley spike: BM-204 Auto Nextseq
SAMN44993558	PRJNA1190462	SRR31505508	Amplicon of barley spike: BM-204n Auto Miseq
SAMN44993558	PRJNA1190462	SRR31505496	Amplicon of barley spike: BM-204o Man Miseq
SAMN44993558	PRJNA1190462	SRR31505581	Amplicon of barley spike: BM-213 Auto Nextseq
SAMN44993558	PRJNA1190462	SRR31505570	Amplicon of barley spike: BM-213n Auto Miseq
SAMN44993558	PRJNA1190462	SRR31505415	Amplicon of barley spike: BM-213o Man Miseq
SAMN44993558	PRJNA1190462	SRR31505404	Amplicon of barley spike: BM-214 Auto Nextseq
SAMN44993558	PRJNA1190462	SRR31505393	Amplicon of barley spike: BM-214n Auto Miseq
SAMN44993558	PRJNA1190462	SRR31505382	Amplicon of barley spike: BM-214o Man Miseq
SAMN44993558	PRJNA1190462	SRR31505659	Amplicon of barley spike: BM-218 Auto Nextseq
SAMN44993558	PRJNA1190462	SRR31505648	Amplicon of barley spike: BM-218n Auto Miseq
SAMN44993558	PRJNA1190462	SRR31505639	Amplicon of barley spike: BM-218o Man Miseq
SAMN44993558	PRJNA1190462	SRR31505637	Amplicon of barley spike: BM-220 Auto Nextseq
SAMN44993558	PRJNA1190462	SRR31505636	Amplicon of barley spike: BM-220n Auto Miseq
SAMN44993558	PRJNA1190462	SRR31505635	Amplicon of barley spike: BM-220o Man Miseq
SAMN44993558	PRJNA1190462	SRR31505634	Amplicon of barley spike: BM-224 Auto Nextseq
SAMN44993558	PRJNA1190462	SRR31505561	Amplicon of barley spike: BM-224n Auto Miseq
SAMN44993558	PRJNA1190462	SRR31505560	Amplicon of barley spike: BM-224o Man Miseq
SAMN44993558	PRJNA1190462	SRR31505559	Amplicon of barley spike: BM-225 Auto Nextseq

SAMN44993558	PRJNA1190462	SRR31505558	Amplicon of barley spike: BM-225n Auto Miseq
SAMN44993558	PRJNA1190462	SRR31505557	Amplicon of barley spike: BM-225o Man Miseq
SAMN44993558	PRJNA1190462	SRR31505556	Amplicon of barley spike: BM-228 Auto Nextseq
SAMN44993558	PRJNA1190462	SRR31505554	Amplicon of barley spike: BM-228n Auto Miseq
SAMN44993558	PRJNA1190462	SRR31505553	Amplicon of barley spike: BM-228o Man Miseq
SAMN44993558	PRJNA1190462	SRR31505552	Amplicon of barley spike: BM-231 Auto Nextseq
SAMN44993558	PRJNA1190462	SRR31505551	Amplicon of barley spike: BM-231n Auto Miseq
SAMN44993558	PRJNA1190462	SRR31505550	Amplicon of barley spike: BM-231o Man Miseq
SAMN44993558	PRJNA1190462	SRR31505549	Amplicon of barley spike: BM-232 Auto Nextseq
SAMN44993558	PRJNA1190462	SRR31505548	Amplicon of barley spike: BM-232n Auto Miseq
SAMN44993558	PRJNA1190462	SRR31505547	Amplicon of barley spike: BM-232o Man Miseq
SAMN44993558	PRJNA1190462	SRR31505546	Amplicon of barley spike: BM-233 Auto Nextseq
SAMN44993558	PRJNA1190462	SRR31505545	Amplicon of barley spike: BM-233n Auto Miseq
SAMN44993558	PRJNA1190462	SRR31505543	Amplicon of barley spike: BM-233o Man Miseq
SAMN44993558	PRJNA1190462	SRR31505542	Amplicon of barley spike: BM-234 Auto Nextseq
SAMN44993558	PRJNA1190462	SRR31505541	Amplicon of barley spike: BM-234n Auto Miseq
SAMN44993558	PRJNA1190462	SRR31505540	Amplicon of barley spike: BM-234o Man Miseq
SAMN44993558	PRJNA1190462	SRR31505539	Amplicon of barley spike: BM-235 Auto Nextseq
SAMN44993558	PRJNA1190462	SRR31505538	Amplicon of barley spike: BM-235n Auto Miseq
SAMN44993558	PRJNA1190462	SRR31505489	Amplicon of barley spike: BM-235o Man Miseq
SAMN44993558	PRJNA1190462	SRR31505488	Amplicon of barley spike: BM-237 Auto Nextseq
SAMN44993558	PRJNA1190462	SRR31505487	Amplicon of barley spike: BM-237n Auto Miseq
SAMN44993558	PRJNA1190462	SRR31505486	Amplicon of barley spike: BM-237o Man Miseq
SAMN44993558	PRJNA1190462	SRR31505484	Amplicon of barley spike: BM-240 Auto Nextseq
SAMN44993558	PRJNA1190462	SRR31505483	Amplicon of barley spike: BM-240n Auto Miseq

SAMN44993558	PRJNA1190462	SRR31505482	Amplicon of barley spike: BM-240o Man Miseq
SAMN44993558	PRJNA1190462	SRR31505481	Amplicon of barley spike: BM-241 Auto Nextseq
SAMN44993558	PRJNA1190462	SRR31505480	Amplicon of barley spike: BM-241n Auto Miseq
SAMN44993558	PRJNA1190462	SRR31505479	Amplicon of barley spike: BM-241o Man Miseq
SAMN44993558	PRJNA1190462	SRR31505478	Amplicon of barley spike: BM-244 Auto Nextseq
SAMN44993558	PRJNA1190462	SRR31505477	Amplicon of barley spike: BM-244n Auto Miseq
SAMN44993558	PRJNA1190462	SRR31505476	Amplicon of barley spike: BM-244o Man Miseq
SAMN44993558	PRJNA1190462	SRR31505475	Amplicon of barley spike: BM-249 Auto Nextseq
SAMN44993558	PRJNA1190462	SRR31505473	Amplicon of barley spike: BM-249n Auto Miseq
SAMN44993558	PRJNA1190462	SRR31505472	Amplicon of barley spike: BM-249o Man Miseq
SAMN44993558	PRJNA1190462	SRR31505471	Amplicon of barley spike: BM-251 Auto Nextseq
SAMN44993558	PRJNA1190462	SRR31505470	Amplicon of barley spike: BM-251n Auto Miseq
SAMN44993558	PRJNA1190462	SRR31505469	Amplicon of barley spike: BM-251o Man Miseq
SAMN44993558	PRJNA1190462	SRR31505468	Amplicon of barley spike: BM-254 Auto Nextseq
SAMN44993558	PRJNA1190462	SRR31505467	Amplicon of barley spike: BM-254n Auto Miseq
SAMN44993558	PRJNA1190462	SRR31505466	Amplicon of barley spike: BM-254o Man Miseq
SAMN44993558	PRJNA1190462	SRR31505441	Amplicon of barley spike: BM-26 Auto Nextseq
SAMN44993558	PRJNA1190462	SRR31505440	Amplicon of barley spike: BM-26n Auto Miseq
SAMN44993558	PRJNA1190462	SRR31505438	Amplicon of barley spike: BM-26o Man Miseq
SAMN44993558	PRJNA1190462	SRR31505437	Amplicon of barley spike: BM-261 Auto Nextseq
SAMN44993558	PRJNA1190462	SRR31505436	Amplicon of barley spike: BM-261n Auto Miseq
SAMN44993558	PRJNA1190462	SRR31505435	Amplicon of barley spike: BM-261o Man Miseq
SAMN44993558	PRJNA1190462	SRR31505434	Amplicon of barley spike: BM-268 Auto Nextseq
SAMN44993558	PRJNA1190462	SRR31505433	Amplicon of barley spike: BM-268n Auto Miseq
SAMN44993558	PRJNA1190462	SRR31505432	Amplicon of barley spike: BM-268o Man Miseq

SAMN44993558	PRJNA1190462	SRR31505431	Amplicon of barley spike: BM-271 Auto Nextseq
SAMN44993558	PRJNA1190462	SRR31505430	Amplicon of barley spike: BM-271n Auto Miseq
SAMN44993558	PRJNA1190462	SRR31505429	Amplicon of barley spike: BM-271o Man Miseq
SAMN44993558	PRJNA1190462	SRR31505427	Amplicon of barley spike: BM-273 Auto Nextseq
SAMN44993558	PRJNA1190462	SRR31505426	Amplicon of barley spike: BM-273n Auto Miseq
SAMN44993558	PRJNA1190462	SRR31505425	Amplicon of barley spike: BM-273o Man Miseq
SAMN44993558	PRJNA1190462	SRR31505424	Amplicon of barley spike: BM-274 Auto Nextseq
SAMN44993558	PRJNA1190462	SRR31505423	Amplicon of barley spike: BM-274n Auto Miseq
SAMN44993558	PRJNA1190462	SRR31505422	Amplicon of barley spike: BM-274o Man Miseq
SAMN44993558	PRJNA1190462	SRR31505421	Amplicon of barley spike: BM-277 Auto Nextseq
SAMN44993558	PRJNA1190462	SRR31505420	Amplicon of barley spike: BM-277n Auto Miseq
SAMN44993558	PRJNA1190462	SRR31505419	Amplicon of barley spike: BM-277o Man Miseq
SAMN44993558	PRJNA1190462	SRR31505418	Amplicon of barley spike: BM-282 Auto Nextseq
SAMN44993558	PRJNA1190462	SRR31505630	Amplicon of barley spike: BM-282n Auto Miseq
SAMN44993558	PRJNA1190462	SRR31505629	Amplicon of barley spike: BM-282o Man Miseq
SAMN44993558	PRJNA1190462	SRR31505628	Amplicon of barley spike: BM-284 Auto Nextseq
SAMN44993558	PRJNA1190462	SRR31505627	Amplicon of barley spike: BM-284n Auto Miseq
SAMN44993558	PRJNA1190462	SRR31505626	Amplicon of barley spike: BM-284o Man Miseq
SAMN44993558	PRJNA1190462	SRR31505625	Amplicon of barley spike: BM-287 Auto Nextseq
SAMN44993558	PRJNA1190462	SRR31505624	Amplicon of barley spike: BM-287n Auto Miseq
SAMN44993558	PRJNA1190462	SRR31505623	Amplicon of barley spike: BM-287o Man Miseq
SAMN44993558	PRJNA1190462	SRR31505622	Amplicon of barley spike: BM-288 Auto Nextseq
SAMN44993558	PRJNA1190462	SRR31505621	Amplicon of barley spike: BM-288n Auto Miseq
SAMN44993558	PRJNA1190462	SRR31505619	Amplicon of barley spike: BM-288o Man Miseq
SAMN44993558	PRJNA1190462	SRR31505618	Amplicon of barley spike: BM-290 Auto Nextseq

SAMN44993558	PRJNA1190462	SRR31505617	Amplicon of barley spike: BM-290n Auto Miseq
SAMN44993558	PRJNA1190462	SRR31505616	Amplicon of barley spike: BM-290o Man Miseq
SAMN44993558	PRJNA1190462	SRR31505615	Amplicon of barley spike: BM-291 Auto Nextseq
SAMN44993558	PRJNA1190462	SRR31505614	Amplicon of barley spike: BM-291n Auto Miseq
SAMN44993558	PRJNA1190462	SRR31505613	Amplicon of barley spike: BM-291o Man Miseq
SAMN44993558	PRJNA1190462	SRR31505612	Amplicon of barley spike: BM-292 Auto Nextseq
SAMN44993558	PRJNA1190462	SRR31505611	Amplicon of barley spike: BM-292n Auto Miseq
SAMN44993558	PRJNA1190462	SRR31505610	Amplicon of barley spike: BM-292o Man Miseq
SAMN44993558	PRJNA1190462	SRR31505608	Amplicon of barley spike: BM-293 Auto Nextseq
SAMN44993558	PRJNA1190462	SRR31505607	Amplicon of barley spike: BM-293n Auto Miseq
SAMN44993558	PRJNA1190462	SRR31505606	Amplicon of barley spike: BM-293o Man Miseq
SAMN44993558	PRJNA1190462	SRR31505605	Amplicon of barley spike: BM-299 Auto Nextseq
SAMN44993558	PRJNA1190462	SRR31505604	Amplicon of barley spike: BM-299n Auto Miseq
SAMN44993558	PRJNA1190462	SRR31505603	Amplicon of barley spike: BM-299o Man Miseq
SAMN44993558	PRJNA1190462	SRR31505602	Amplicon of barley spike: BM-3 Auto Nextseq
SAMN44993558	PRJNA1190462	SRR31505601	Amplicon of barley spike: BM-3n Auto Miseq
SAMN44993558	PRJNA1190462	SRR31505600	Amplicon of barley spike: BM-3o Man Miseq
SAMN44993558	PRJNA1190462	SRR31505599	Amplicon of barley spike: BM-302 Auto Nextseq
SAMN44993558	PRJNA1190462	SRR31505597	Amplicon of barley spike: BM-302n Auto Miseq
SAMN44993558	PRJNA1190462	SRR31505596	Amplicon of barley spike: BM-302o Man Miseq
SAMN44993558	PRJNA1190462	SRR31505595	Amplicon of barley spike: BM-304 Auto Nextseq
SAMN44993558	PRJNA1190462	SRR31505594	Amplicon of barley spike: BM-304n Auto Miseq
SAMN44993558	PRJNA1190462	SRR31505593	Amplicon of barley spike: BM-304o Man Miseq
SAMN44993558	PRJNA1190462	SRR31505592	Amplicon of barley spike: BM-305 Auto Nextseq
SAMN44993558	PRJNA1190462	SRR31505591	Amplicon of barley spike: BM-305n Auto Miseq

SAMN44993558	PRJNA1190462	SRR31505590	Amplicon of barley spike: BM-305o Man Miseq
SAMN44993558	PRJNA1190462	SRR31505589	Amplicon of barley spike: BM-307 Auto Nextseq
SAMN44993558	PRJNA1190462	SRR31505588	Amplicon of barley spike: BM-307n Auto Miseq
SAMN44993558	PRJNA1190462	SRR31505586	Amplicon of barley spike: BM-307o Man Miseq
SAMN44993558	PRJNA1190462	SRR31505537	Amplicon of barley spike: BM-309 Auto Nextseq
SAMN44993558	PRJNA1190462	SRR31505536	Amplicon of barley spike: BM-309n Auto Miseq
SAMN44993558	PRJNA1190462	SRR31505535	Amplicon of barley spike: BM-309o Man Miseq
SAMN44993558	PRJNA1190462	SRR31505534	Amplicon of barley spike: BM-31 Auto Nextseq
SAMN44993558	PRJNA1190462	SRR31505533	Amplicon of barley spike: BM-31n Auto Miseq
SAMN44993558	PRJNA1190462	SRR31505532	Amplicon of barley spike: BM-31o Man Miseq
SAMN44993558	PRJNA1190462	SRR31505531	Amplicon of barley spike: BM-312 Auto Nextseq
SAMN44993558	PRJNA1190462	SRR31505530	Amplicon of barley spike: BM-312n Auto Miseq
SAMN44993558	PRJNA1190462	SRR31505529	Amplicon of barley spike: BM-312o Man Miseq
SAMN44993558	PRJNA1190462	SRR31505527	Amplicon of barley spike: BM-313 Auto Nextseq
SAMN44993558	PRJNA1190462	SRR31505526	Amplicon of barley spike: BM-313n Auto Miseq
SAMN44993558	PRJNA1190462	SRR31505525	Amplicon of barley spike: BM-313o Man Miseq
SAMN44993558	PRJNA1190462	SRR31505524	Amplicon of barley spike: BM-314 Auto Nextseq
SAMN44993558	PRJNA1190462	SRR31505523	Amplicon of barley spike: BM-314n Auto Miseq
SAMN44993558	PRJNA1190462	SRR31505522	Amplicon of barley spike: BM-314o Man Miseq
SAMN44993558	PRJNA1190462	SRR31505521	Amplicon of barley spike: BM-315 Auto Nextseq
SAMN44993558	PRJNA1190462	SRR31505520	Amplicon of barley spike: BM-315n Auto Miseq
SAMN44993558	PRJNA1190462	SRR31505519	Amplicon of barley spike: BM-315o Man Miseq
SAMN44993558	PRJNA1190462	SRR31505518	Amplicon of barley spike: BM-320 Auto Nextseq
SAMN44993558	PRJNA1190462	SRR31505516	Amplicon of barley spike: BM-320n Auto Miseq
SAMN44993558	PRJNA1190462	SRR31505515	Amplicon of barley spike: BM-320o Man Miseq

SAMN44993558	PRJNA1190462	SRR31505514	Amplicon of barley spike: BM-322 Auto Nextseq
SAMN44993558	PRJNA1190462	SRR31505465	Amplicon of barley spike: BM-322n Auto Miseq
SAMN44993558	PRJNA1190462	SRR31505464	Amplicon of barley spike: BM-322o Man Miseq
SAMN44993558	PRJNA1190462	SRR31505463	Amplicon of barley spike: BM-328 Auto Nextseq
SAMN44993558	PRJNA1190462	SRR31505462	Amplicon of barley spike: BM-328n Auto Miseq
SAMN44993558	PRJNA1190462	SRR31505461	Amplicon of barley spike: BM-328o Man Miseq
SAMN44993558	PRJNA1190462	SRR31505460	Amplicon of barley spike: BM-331 Auto Nextseq
SAMN44993558	PRJNA1190462	SRR31505459	Amplicon of barley spike: BM-331n Auto Miseq
SAMN44993558	PRJNA1190462	SRR31505457	Amplicon of barley spike: BM-331o Man Miseq
SAMN44993558	PRJNA1190462	SRR31505456	Amplicon of barley spike: BM-338 Auto Nextseq
SAMN44993558	PRJNA1190462	SRR31505455	Amplicon of barley spike: BM-338n Auto Miseq
SAMN44993558	PRJNA1190462	SRR31505454	Amplicon of barley spike: BM-338o Man Miseq
SAMN44993558	PRJNA1190462	SRR31505453	Amplicon of barley spike: BM-34 Auto Nextseq
SAMN44993558	PRJNA1190462	SRR31505452	Amplicon of barley spike: BM-34n Auto Miseq
SAMN44993558	PRJNA1190462	SRR31505451	Amplicon of barley spike: BM-34o Man Miseq
SAMN44993558	PRJNA1190462	SRR31505450	Amplicon of barley spike: BM-340 Auto Nextseq
SAMN44993558	PRJNA1190462	SRR31505449	Amplicon of barley spike: BM-340n Auto Miseq
SAMN44993558	PRJNA1190462	SRR31505448	Amplicon of barley spike: BM-340o Man Miseq
SAMN44993558	PRJNA1190462	SRR31505446	Amplicon of barley spike: BM-341 Auto Nextseq
SAMN44993558	PRJNA1190462	SRR31505445	Amplicon of barley spike: BM-341n Auto Miseq
SAMN44993558	PRJNA1190462	SRR31505444	Amplicon of barley spike: BM-341o Man Miseq
SAMN44993558	PRJNA1190462	SRR31505443	Amplicon of barley spike: BM-342 Auto Nextseq
SAMN44993558	PRJNA1190462	SRR31505442	Amplicon of barley spike: BM-342n Auto Miseq
SAMN44993558	PRJNA1190462	SRR31505513	Amplicon of barley spike: BM-342o Man Miseq
SAMN44993558	PRJNA1190462	SRR31505512	Amplicon of barley spike: BM-346 Auto Nextseq

SAMN44993558	PRJNA1190462	SRR31505511	Amplicon of barley spike: BM-346n Auto Miseq
SAMN44993558	PRJNA1190462	SRR31505510	Amplicon of barley spike: BM-346o Man Miseq
SAMN44993558	PRJNA1190462	SRR31505509	Amplicon of barley spike: BM-348 Auto Nextseq
SAMN44993558	PRJNA1190462	SRR31505507	Amplicon of barley spike: BM-348n Auto Miseq
SAMN44993558	PRJNA1190462	SRR31505506	Amplicon of barley spike: BM-348o Man Miseq
SAMN44993558	PRJNA1190462	SRR31505505	Amplicon of barley spike: BM-351 Auto Nextseq
SAMN44993558	PRJNA1190462	SRR31505504	Amplicon of barley spike: BM-351n Auto Miseq
SAMN44993558	PRJNA1190462	SRR31505503	Amplicon of barley spike: BM-351o Man Miseq
SAMN44993558	PRJNA1190462	SRR31505502	Amplicon of barley spike: BM-353 Auto Nextseq
SAMN44993558	PRJNA1190462	SRR31505501	Amplicon of barley spike: BM-353n Auto Miseq
SAMN44993558	PRJNA1190462	SRR31505500	Amplicon of barley spike: BM-353o Man Miseq
SAMN44993558	PRJNA1190462	SRR31505499	Amplicon of barley spike: BM-355 Auto Nextseq
SAMN44993558	PRJNA1190462	SRR31505498	Amplicon of barley spike: BM-355n Auto Miseq
SAMN44993558	PRJNA1190462	SRR31505495	Amplicon of barley spike: BM-355o Man Miseq
SAMN44993558	PRJNA1190462	SRR31505494	Amplicon of barley spike: BM-358 Auto Nextseq
SAMN44993558	PRJNA1190462	SRR31505493	Amplicon of barley spike: BM-358n Auto Miseq
SAMN44993558	PRJNA1190462	SRR31505492	Amplicon of barley spike: BM-358o Man Miseq
SAMN44993558	PRJNA1190462	SRR31505491	Amplicon of barley spike: BM-36 Auto Nextseq
SAMN44993558	PRJNA1190462	SRR31505490	Amplicon of barley spike: BM-36n Auto Miseq
SAMN44993558	PRJNA1190462	SRR31505585	Amplicon of barley spike: BM-36o Man Miseq
SAMN44993558	PRJNA1190462	SRR31505584	Amplicon of barley spike: BM-365 Auto Nextseq
SAMN44993558	PRJNA1190462	SRR31505583	Amplicon of barley spike: BM-365n Auto Miseq
SAMN44993558	PRJNA1190462	SRR31505582	Amplicon of barley spike: BM-365o Man Miseq
SAMN44993558	PRJNA1190462	SRR31505580	Amplicon of barley spike: BM-376 Auto Nextseq
SAMN44993558	PRJNA1190462	SRR31505579	Amplicon of barley spike: BM-376n Auto Miseq

SAMN44993558	PRJNA1190462	SRR31505578	Amplicon of barley spike: BM-376o Man Miseq
SAMN44993558	PRJNA1190462	SRR31505577	Amplicon of barley spike: BM-383 Auto Nextseq
SAMN44993558	PRJNA1190462	SRR31505576	Amplicon of barley spike: BM-383n Auto Miseq
SAMN44993558	PRJNA1190462	SRR31505575	Amplicon of barley spike: BM-383o Man Miseq
SAMN44993558	PRJNA1190462	SRR31505574	Amplicon of barley spike: BM-386 Auto Nextseq
SAMN44993558	PRJNA1190462	SRR31505573	Amplicon of barley spike: BM-386n Auto Miseq
SAMN44993558	PRJNA1190462	SRR31505572	Amplicon of barley spike: BM-386o Man Miseq
SAMN44993558	PRJNA1190462	SRR31505571	Amplicon of barley spike: BM-391 Auto Nextseq
SAMN44993558	PRJNA1190462	SRR31505569	Amplicon of barley spike: BM-391n Auto Miseq
SAMN44993558	PRJNA1190462	SRR31505568	Amplicon of barley spike: BM-391o Man Miseq
SAMN44993558	PRJNA1190462	SRR31505567	Amplicon of barley spike: BM-399 Auto Nextseq
SAMN44993558	PRJNA1190462	SRR31505566	Amplicon of barley spike: BM-399n Auto Miseq
SAMN44993558	PRJNA1190462	SRR31505565	Amplicon of barley spike: BM-399o Man Miseq
SAMN44993558	PRJNA1190462	SRR31505564	Amplicon of barley spike: BM-44 Auto Nextseq
SAMN44993558	PRJNA1190462	SRR31505563	Amplicon of barley spike: BM-44n Auto Miseq
SAMN44993558	PRJNA1190462	SRR31505562	Amplicon of barley spike: BM-44o Man Miseq
SAMN44993558	PRJNA1190462	SRR31505417	Amplicon of barley spike: BM-49 Auto Nextseq
SAMN44993558	PRJNA1190462	SRR31505416	Amplicon of barley spike: BM-49n Auto Miseq
SAMN44993558	PRJNA1190462	SRR31505414	Amplicon of barley spike: BM-49o Man Miseq
SAMN44993558	PRJNA1190462	SRR31505413	Amplicon of barley spike: BM-50 Auto Nextseq
SAMN44993558	PRJNA1190462	SRR31505412	Amplicon of barley spike: BM-50n Auto Miseq
SAMN44993558	PRJNA1190462	SRR31505411	Amplicon of barley spike: BM-50o Man Miseq
SAMN44993558	PRJNA1190462	SRR31505410	Amplicon of barley spike: BM-55 Auto Nextseq
SAMN44993558	PRJNA1190462	SRR31505409	Amplicon of barley spike: BM-55n Auto Miseq
SAMN44993558	PRJNA1190462	SRR31505408	Amplicon of barley spike: BM-55o Man Miseq

SAMN44993558	PRJNA1190462	SRR31505407	Amplicon of barley spike: BM-6 Auto Nextseq
SAMN44993558	PRJNA1190462	SRR31505406	Amplicon of barley spike: BM-6n Auto Miseq
SAMN44993558	PRJNA1190462	SRR31505405	Amplicon of barley spike: BM-6o Man Miseq
SAMN44993558	PRJNA1190462	SRR31505403	Amplicon of barley spike: BM-64 Auto Nextseq
SAMN44993558	PRJNA1190462	SRR31505402	Amplicon of barley spike: BM-64n Auto Miseq
SAMN44993558	PRJNA1190462	SRR31505401	Amplicon of barley spike: BM-64o Man Miseq
SAMN44993558	PRJNA1190462	SRR31505400	Amplicon of barley spike: BM-65 Auto Nextseq
SAMN44993558	PRJNA1190462	SRR31505399	Amplicon of barley spike: BM-65n Auto Miseq
SAMN44993558	PRJNA1190462	SRR31505398	Amplicon of barley spike: BM-65o Man Miseq
SAMN44993558	PRJNA1190462	SRR31505397	Amplicon of barley spike: BM-68 Auto Nextseq
SAMN44993558	PRJNA1190462	SRR31505396	Amplicon of barley spike: BM-68n Auto Miseq
SAMN44993558	PRJNA1190462	SRR31505395	Amplicon of barley spike: BM-68o Man Miseq
SAMN44993558	PRJNA1190462	SRR31505394	Amplicon of barley spike: BM-69 Auto Nextseq
SAMN44993558	PRJNA1190462	SRR31505392	Amplicon of barley spike: BM-69n Auto Miseq
SAMN44993558	PRJNA1190462	SRR31505391	Amplicon of barley spike: BM-69o Man Miseq
SAMN44993558	PRJNA1190462	SRR31505390	Amplicon of barley spike: BM-72 Auto Nextseq
SAMN44993558	PRJNA1190462	SRR31505389	Amplicon of barley spike: BM-72n Auto Miseq
SAMN44993558	PRJNA1190462	SRR31505388	Amplicon of barley spike: BM-72o Man Miseq
SAMN44993558	PRJNA1190462	SRR31505387	Amplicon of barley spike: BM-73 Auto Nextseq
SAMN44993558	PRJNA1190462	SRR31505386	Amplicon of barley spike: BM-73n Auto Miseq
SAMN44993558	PRJNA1190462	SRR31505385	Amplicon of barley spike: BM-73o Man Miseq
SAMN44993558	PRJNA1190462	SRR31505384	Amplicon of barley spike: BM-78 Auto Nextseq
SAMN44993558	PRJNA1190462	SRR31505383	Amplicon of barley spike: BM-78n Auto Miseq
SAMN44993558	PRJNA1190462	SRR31505381	Amplicon of barley spike: BM-78o Man Miseq
SAMN44993558	PRJNA1190462	SRR31505380	Amplicon of barley spike: BM-8 Auto Nextseq

SAMN44993558	PRJNA1190462	SRR31505379	Amplicon of barley spike: BM-8n Auto Miseq
SAMN44993558	PRJNA1190462	SRR31505666	Amplicon of barley spike: BM-8o Man Miseq
SAMN44993558	PRJNA1190462	SRR31505665	Amplicon of barley spike: BM-82 Auto Nextseq
SAMN44993558	PRJNA1190462	SRR31505664	Amplicon of barley spike: BM-82n Auto Miseq
SAMN44993558	PRJNA1190462	SRR31505663	Amplicon of barley spike: BM-82o Man Miseq
SAMN44993558	PRJNA1190462	SRR31505662	Amplicon of barley spike: BM-89 Auto Nextseq
SAMN44993558	PRJNA1190462	SRR31505661	Amplicon of barley spike: BM-89n Auto Miseq
SAMN44993558	PRJNA1190462	SRR31505660	Amplicon of barley spike: BM-89o Man Miseq
SAMN44993558	PRJNA1190462	SRR31505658	Amplicon of barley spike: BM-92 Auto Nextseq
SAMN44993558	PRJNA1190462	SRR31505657	Amplicon of barley spike: BM-92n Auto Miseq
SAMN44993558	PRJNA1190462	SRR31505656	Amplicon of barley spike: BM-92o Man Miseq
SAMN44993558	PRJNA1190462	SRR31505655	Amplicon of barley spike: BM-93 Auto Nextseq
SAMN44993558	PRJNA1190462	SRR31505654	Amplicon of barley spike: BM-93n Auto Miseq
SAMN44993558	PRJNA1190462	SRR31505653	Amplicon of barley spike: BM-93o Man Miseq
SAMN44993558	PRJNA1190462	SRR31505652	Amplicon of barley spike: BM-98 Auto Nextseq
SAMN44993558	PRJNA1190462	SRR31505651	Amplicon of barley spike: BM-98n Auto Miseq
SAMN44993558	PRJNA1190462	SRR31505650	Amplicon of barley spike: BM-98o Man Miseq
SAMN44993558	PRJNA1190462	SRR31505649	Amplicon of barley spike: Ex Neg Ctrl Auto Nextseq
SAMN44993558	PRJNA1190462	SRR31505647	Amplicon of barley spike: Ex Neg Ctrln Auto Miseq
SAMN44993558	PRJNA1190462	SRR31505646	Amplicon of barley spike: Ex Neg Ctrlo Man Miseq
SAMN44993558	PRJNA1190462	SRR31505645	Amplicon of barley spike: PCR Neg Ctrl Auto Nextseq
SAMN44993558	PRJNA1190462	SRR31505644	Amplicon of barley spike: PCR Neg Ctrln Auto Miseq
SAMN44993558	PRJNA1190462	SRR31505643	Amplicon of barley spike: PCR Neg Ctrlo Man Miseq

SAMN44993558	PRJNA1190462	SRR31505642	Amplicon of barley spike: Pos Ctrl Auto Nextseq
SAMN44993558	PRJNA1190462	SRR31505641	Amplicon of barley spike: Pos Ctrln Auto Miseq
SAMN44993558	PRJNA1190462	SRR31505640	Amplicon of barley spike: Pos Ctrlo Man Miseq

Supplementary Table 2. SRA accession numbers
